# Genome-wide binding studies reveal DNA binding specificity mechanisms and functional interplay amongst Forkhead transcription factors

**DOI:** 10.1093/nar/gkv1120

**Published:** 2015-11-17

**Authors:** Xi Chen, Zongling Ji, Aaron Webber, Andrew D. Sharrocks

**Affiliations:** Faculty of Life Sciences, University of Manchester, Michael Smith Building, Oxford Road, Manchester M13 9PT, UK

## Abstract

Transcription factors belonging to the same transcription factor families contain very similar DNA binding domains and hence have the potential to bind to related DNA sequences. However, subtle differences in binding specificities can be detected *in vitro* with the potential to direct specific responses *in vivo*. Here, we have examined the binding properties of three Forkhead (FOX) transcription factors, FOXK2, FOXO3 and FOXJ3 *in vivo*. Extensive overlap in chromatin binding is observed, although underlying differential DNA binding specificity can dictate the recruitment of FOXK2 and FOXJ3 to chromatin. However, functionally, FOXO3-dependent gene regulation is generally mediated not through uniquely bound regions but through regions occupied by both FOXK2 and FOXO3 where both factors play a regulatory role. Our data point to a model whereby FOX transcription factors control gene expression through dynamically binding and generating partial occupancy of the same site rather than mutually exclusive binding derived by stable binding of individual FOX proteins.

## INTRODUCTION

Sequence-specific transcription factors are grouped into families based on the identities of their DNA binding domains. In mammalian systems, these families can consist of dozens of members as exemplified by the Forkhead (FOX) transcription factor family which contains over 50 different proteins in humans (1,2). Due to the high sequence conservation within their DNA binding domains, the intrinsic DNA binding specificities of individual family members are often very similar, and in many cases virtually identical (3,4), creating potential problems for generating specific functional responses at any given site. Indeed, many ChIP-seq studies have demonstrated extensive overlap in the binding of different family members to the same genomic regions, as exemplified by the ETS transcription factor family (5,6). One conclusion arising from these types of studies is that the regions uniquely bound by an individual family member are likely to represent the more functionally relevant sites, and that additional features of these regulatory regions drive this functional specificity. For example, ETS1 interactions with RUNX transcription factors drive specific gene expression responses in T cells (6). Conversely, regions associated with the binding of multiple family members are thought to either be functionally neutral or be involved in maintaining the expression of housekeeping genes. However, these studies illustrate that we still have an incomplete understanding of how transcription factors achieve functional specificity when confronted with a large number of possible binding sites and related proteins competing for binding site occupancy (7).

In this study, we have investigated how FOX transcription factors generate chromatin binding and functional specificity. This family of transcription factors generally bind to DNA through sequences related to the RYAAAYA motif (where R = purine and Y = pyrimidine). We have focused on three widely expressed FOX transcription factors, FOXK2, FOXO3 and FOXJ3. FOXJ3 has previously been shown to have a potential role in cell cycle control (8) and has also been implicated in muscle biology where it is required for myofiber identity and muscle regeneration (9). The biological role of FOXK2 is unclear although links to CDKs suggest a functional connection to the cell cycle (10) and functionally, FOXK2 can interact with and recruit chromatin remodelling complexes (11,12). FOXO3 has been more widely studied and has been implicated in a wide variety of biological roles, including being linked with promoting autophagy in response to cellular starvation and driving apoptotic gene expression programmes (13).

Here, we have examined the genome-wide binding profiles of FOXK2, FOXO3 and FOXJ3 and for direct comparison we used a single cell type. We demonstrate that although each FOX protein has a unique binding profile, we find extensive overlap in the genome-wide binding profiles for FOXK2, FOXO3 and FOXJ3. We identify underlying differences in DNA sequence preferences that helps explain some of the differences in their binding profiles. However, we find that rather than uniquely bound regions, the regions commonly bound by two or more FOX proteins are functionally important for FOXO3-mediated transcriptional activation events. We propose a model where different dynamically binding FOX transcription factors combine to control the expression of target genes.

## MATERIALS AND METHODS

### Plasmid constructs

To generate a plasmid encoding 3xFLAG-FOXO3 (pAS3012), the primer pair ADS3561/ADS3562 was used to introduce BamHI and NotI to the 5′ and 3′ ends of FOXO3 coding sequence, and the PCR product was inserted into a pcDNA5 vector which contains triple FLAG tags at the N-terminal insertion site (pAS3011; gift from Catherine Millar). The same strategy was used to generate a plasmid encoding 3xFLAG-FOXJ3 (pAS3088) using the primer pair ADS3661/ADS3662.

The FOXK2-Sso7d fusion protein expression vector pAS4314 (pCDNA5-FRT-TO-3xFLAG-FOXK2(1–430)-Sso7d(E35L)) was constructed by a two-step procedure. First, FOXK2(1–430) and Sso7dE35L were amplified using the primer–template combinations ADS1305/ADS2531-pAS2252 and ADS4644/ADS2724-pAS4309 (pCTCON-Sso7dE35L; 14), respectively, and digested using XbaI followed by ligation. Next, the fusion PCR product was amplified using primer pair ADS1305/ADS2724 and digested by BamHI before cloning into BamHI and NotI (filled in by treatment with Klenow) sites in pAS3012 (pCDNA5-FRT-TO-3xFLAG-FOXO3) to create pAS4314. pAS3075 encoding 6XHis-tagged-FOXK2(228–382) was generated using the primer pair ADS3637/ADS3638 to introduce NdeI and XhoI sites to the 5′ and 3′ ends of the FOXK2 DNA binding domain coding sequence. Then the PCR product was inserted into a pET3a-Tr. The same strategy was used to generate the expression vector pAS3076 (encoding 6XHis-tagged-FOXO3(130–271)) using the primer pair ADS3639/ADS3640.

All the constructs were verified by sequencing.

### Tissue culture, cell transfection, immunocytochemistry and RNA interference

U2OS cells, were grown in DMEM supplemented with 10% foetal bovine serum. U2OS cells stably expressing FOXK2-HF (U2OS-FOXK2-HF), a control ‘empty vector’ cell line (U2OS-HF) were made and propagated as described previously (10). To create U2OS cell lines stably expressing 3xFLAG-FOXO3 (U2OS-3xFLAG-FOXO3), 3xFLAG-FOXJ3 (U2OS-3xFLAG-FOXJ3), or 3xFLAG-FOXK2(1–430)-Sso7dE35L (U2OS-3xFLAG-FOXK2(1–430)-Sso7d(E35L)) the parental line U2OS-TREX (gift from C Millar), was transfected with pOG44 (Invitrogen) together with either pAS3012 (3xFLAG-FOXO3), pAS3088 (3xFLAG-FOXJ3) or pAS4314 (3xFLAG-FOXK2(1–430)-Sso7dE35L), respectively. Hygromycin-resistant colonies were pooled and expanded.

siRNA against *FOXK2* and a matched Non-targeting (NT) control, were obtained from Dharmacon and RNA interference (RNAi) was performed as described previously (15).

For visualization of FOXO3 subcellular localization, fluorescence microscopy was performed mainly as described previously (10) using these following antibodies: FLAG M2 (Sigma Aldrich, F3165), FOXO3 (75D8) (Cell Signalling), Alexa Fluor 594 donkey anti-mouse (Invitrogen) and Alexa Fluor 488 donkey anti-rabbit (Invitrogen).

### Western blot analysis

Western blotting was carried out with the primary antibodies; FOXK2 (ILF1, Bethyl Laboratories A301–729A), FOXO3 (75D8, Cell Signalling, #2497 or Millipore 07–702), FLAG (Sigma-Aldrich, F3165), ERK2 (sc-154, Santa Cruz), LMNB1 (C-20 Santa Cruz SC-6216) and FOXJ3 (Bethyl Laboratories A303–107). The proteins were detected using Infrared IRDye-labelled secondary antibodies and the signal was collected with a Li-Cor Odyssey infrared imager.

### RT–PCR and expression microarray analysis

mRNA was isolated and real-time RT–PCR was performed essentially as described previously (16). The primer-pairs used for RT–PCR experiments are listed in Supplementary Table S1. For knockdown experiments, RNAi treatment was performed for 48 h before harvesting. For microarray analysis, mRNA labelling and expression profiling using Affymetrix arrays (Human Genome U133 Plus 2.0 Array) was performed and data analysed as described previously (15). Array experiments were performed in duplicate for each experimental condition (i.e. left untreated or treated with 50 μM LY294002 for 2 h). Gene expression changes were considered significant if the changes were >1.5 or <−1.5-fold and had a FDR < 0.05. Experimental data are deposited in array express; E-MTAB-3763.

### His-tagged protein purification and *in vitro* DNA binding assays

BL21-CodonPlus-RIL bacteria (*E. coli* B *F- ompT hsdS(rB- mB-) dcm+ Tet^r^ gal λ endA Hte [argU ileY leuW Cam^r^*]) were transformed with pAS3075 (encoding 6XHis tagged-FOXK2(228–382)) or pAS3076 (encoding 6XHis tagged FOXO3(130–271)). Transformed bacteria were grown at 37°C and at OD_600_ 0.5–0.7, 0.5 mM IPTG was added, and further incubated at 25°C for 4 h. Proteins were then purified using Ni-agarose beads (Qiagen) and the final elutes were dialysed against 1x PBS overnight. After the dialysis, glycerol was added to a final concentration of 30% (v/v), and proteins were stored at −80°C.

Protein–DNA interaction band shift assays were carried out as described previously (17) using DNA duplexes created by annealing the following oligonucleotide sequences surrounding the FOX binding motif (underlined) associated with the *CYP27C1* locus; ADS2782 (5′- CTAGAACATGTTAATGTAAACAAGGAAGCCTG-3′) and ADS2783 (5′-CTAG CAGGCTTCCTTGTTTACATTAACATGTT-3′). Binding reactions with labelled wild-type DNA duplexes were performed in the presence of 75 mM KCl and the appropriate amount of unlabelled competitor DNAs (0 to 200-fold excess); *CYP27C1*mut1 or *CYP27C1*mut2 (Supplementary Table S1). The reaction was incubated at room temperature for 30 min before loading onto a 5% polyacrylamide gel in 0.25x TBE running buffer at 4°C. Dried gels were exposed to phosphorimager screens and the signals were quantified by Quantity One software (Bio-Rad).

### Chromatin immunoprecipitation (ChIP) assays

ChIP assays using control IgG (Millipore 12–370), anti-Flag (Sigma-Aldrich, F3165) or antisera specific to FOXK2 (Bethyl Laboratories A301–729A), FOXO3 (Millipore 07–702), SRF (Santa Cruz SC-335X) or ELK1 (Epitomics 1277–1) were performed as described previously (10).

Bound regions were detected by quantitative PCR (using primers listed in Supplementary Table S1), at least in duplicate, from at least two independent experiments, using Quantitect SYBR green PCR reagent (Qiagen). Results were analysed with Rotorgene Q series software (Qiagen) relative to input using the standard curve method.

### ChIP-seq assays

ChIP was performed as previously described (15) using ∼8–10 × 10^7^ U2OS (H3K18 acetylation), U2OS-3xFLAG-FOXO3 or U2OS-3xFLAG-FOXJ3 cells stably expressing FOXO3 and FOXJ3, respectively, tagged with 3xFLAG tags. For ChIP-seq, ∼10–20 ng of immunoprecipitated DNA was sent for sequencing using the Illumina Hiseq2000 platform according to the manufacturer's protocols. Experimental data are deposited in array express; E-MTAB-3687 (FOXJ3), E-MTAB-3695 (histone H3K18 acetylation) E-MTAB-2204 (FOXK2) and E-MTAB-2701 (FOXO3).

### Bioinformatics analysis

For ChIP-seq analyses, the first 50 bp of the raw reads were mapped to hg18 using bowtie (18) with default setting, except that ‘−m1’ option was specified which only keeps reads that can be uniquely mapped to the genome. The number of reads was: 8 231 411 (FOXK2), 26 174 803 (FOXO3), 31 867 045 (FOXJ3) and 24 792 957 (H3K18ac). The binding regions were identified by peak calling using MACS 1.4.2 (19) and HOMER version 4.7 (20), with fragment size set as 200 bp. Both programs were used using default thresholds (MACS; *P* < 1e^−5^, and all the peaks returned have a FDR < 5.67% and HOMER; P < 1e^−4^ and a 4-fold enrichment over input). Peaks that were identified by both peak callers were retained and MACS coordinates were used for downstream analysis. Peaks that have at least 1 bp in common were considered as overlapping, and the mergePeak functionality from HOMER was used in this case. Enriched motifs were identified by findMotifsGenome.pl from HOMER using 200 bp spanning the summit of the transcription factor binding region.

To associate binding peaks to genes and find the distance between two peaks, annotatePeak.pl from HOMER was used (20). The peak was assigned to a gene only if the summit is within between −5k and +2k from its transcriptional start site. HOMER (20) was also used to identify gene ontology (GO) categories associated with different categories of genes whose regulatory regions are bound by FOX proteins.

To identify base preferences flanking the core Forkhead motif GTAAACA, the sequences located up to 5 bp upstream and downstream of these motifs were extracted. GTAAACA motifs found in the 200 bp regions flanking FOXK2 and FOXO3 summits were compared to the same number of GTAAACA motifs taken from the whole genome. The number of A, C, G and T at each flanking position was counted, the process repeated 1000 times on the whole genome (simulation), and the average and standard deviation of A, C, G and T at each position were calculated. If the occurrence of a particular base at a flanking position is more than 13 standard deviations than the simulation, it was considered as significant.

## RESULTS

### Genome-wide identification of the FOXK2 and FOXO3 binding regions

To begin to probe the *in vivo* DNA binding properties of FOX transcription factors we first investigated FOXK2 and compared this with the extensively studied FOX protein, FOXO3. Both of these proteins are ubiquitously expressed and are among the highest expressed FOX proteins in U2OS cells (Supplementary Figure S1). Previously we identified regions in the genome occupied by FOXK2 *in vivo* by performing ChIP-seq with an anti-Flag antibody in a (U2OS)-derived cell line which stably expresses FOXK2 fused to a hexahistidine and triple flag tag (U2OS-FOXK2-HF cells) (15). To compare the genome-wide binding profile of FOXO3 under comparable conditions, we used the same strategy and created a (U2OS)-derived cell line which stably expresses FOXO3 fused to a triple FLAG tag (U2OS-3xFLAG-FOXO3 cells). In this case we expressed 3xFLAG-FOXO3 under the control of a doxycycline inducible promoter and determined the dosage of doxycycline which expressed 3xFLAG-FOXO3 at similar levels to the endogenous protein (Supplementary Figure S2A). Importantly, 3xFLAG-FOXO3 appeared functionally normal as it translocated to the nucleus following inhibition of phosphatidylinositol 3-kinase (PI3K) by treatment with LY294002 and inducible binding was detected on target gene promoters *PAQR8* and *CYP27C1* (Supplementary Figure S2B and C). ChIP was then performed on U2OS-3xFLAG-FOXO3 cells following treatment with LY294002, and the precipitated material sequenced using the Illumina platform (ChIP-seq). We analysed the FOXO3 and reanalysed the FOXK2 ChIP-seq data by identifying high confidence binding regions that were generated by two different peak callers; MACS (19) and HOMER (20). This generated 8966 (for FOXO3) and 40 537 (for FOXK2) binding regions. Importantly, we were able to validate a large number of the FOXO3 binding regions by ChIP-qPCR (Supplementary Figure S3A) and the expected Gene Ontology terms associated with apoptotic control were identified among the FOXO3 target genes (Supplementary Figure S3B). Furthermore, the top scoring motif identified in the FOXO3 binding regions corresponded to a FOX binding motif (Figure 2A), providing further validation of the success of this ChIP-seq experiment.

**Figure 1.**
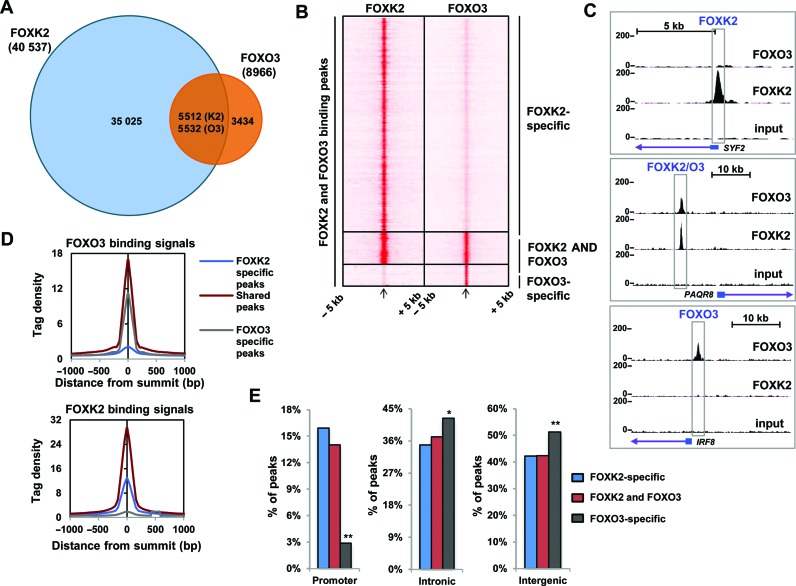
ChIP-seq analysis of FOXK2 and FOXO3 binding profiles. (**A**) Venn diagram showing overlapping binding regions shared between FOXK2 and FOXO3. Numbers of peaks overlapping with respect to FOXK2 (K2) and FOXO3 (O3) are shown. (**B**) Heat map of tag densities of FOXK2 (left) or FOXO3 (right) ChIP-seq signal at all of the binding regions identified in the FOXK2 and FOXO3 ChIP-seq experiments. In each heat map the tag density is plotted for 5 kb either side of its binding peak summit. (**C**) UCSC genome browser views of FOXK2 and FOXO3 binding profiles associated with the indicated loci. (**D**) Average tag density profiles of FOXO3 (top) and FOXK2 (bottom) analyses mapped onto binding region summits of the indicated peak categories. (**E**) Genomic locations of the indicated groups of FOXK2 and FOXO3 binding regions. * = *P* < 0.05; **P < 0.01.

Next we determined the overlap in FOXK2 and FOXO3 binding regions and unexpectedly, we found that 62% of the FOXO3 binding regions were also occupied by FOXK2 (Figure 1A). Three distinct categories of binding regions could be detected; in addition to the regions bound by both FOXO3 and FOXK2 (termed FOXK2/O3), the largest number were preferentially bound by FOXK2 (termed FOXK2-specific), while relatively few were preferentially bound by FOXO3 (termed FOXO3-specific) (Figure 1B and C). When we compared the ChIP-seq tag density around FOXO3 binding sites, this was similar in FOXK2/O3 shared and FOXO3-specific peaks. In contrast, the FOXK2 tag density was substantially higher in the FOXK2/O3 shared peaks than the FOXK2-specific peaks (Figure 1D). Thus, there seems to be no preferential higher levels of binding to regions specifically bound by a particular FOX transcription factor, rather the shared regions showed the more robust binding of each factor. Next we asked whether there was any preference for binding at any particular genomic location for the different categories of FOX target genes and found that FOXO3-specific genes were more likely to fall in intronic and intergenic regions compared to the FOXK2/O3 shared and FOXK2-specific regions which instead were more common in promoter regions (Figure 1E).

Together these data demonstrate widespread binding of FOXO3 and FOXK2 to the same genomic locations, and these shared FOXK2/O3 binding regions are strongly bound by both FOX proteins.

### DNA sequence determinants for FOXK2 and FOXO3 binding

**Figure 2.**
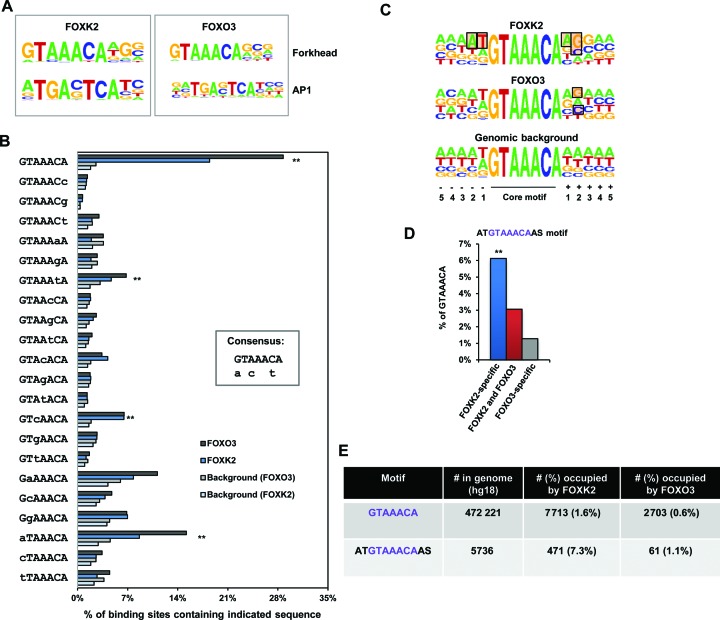
Sequence motifs found in the FOXK2 and FOXO3 binding regions. (**A**) WebLogo representation of over-represented motifs identified by de novo motif discovery in the FOXK2 (left) and FOXO3 (right) binding regions. (**B**) Binding specificity of FOXK2. The occurrence of each of the indicated motifs in the FOXO3 or FOXK2 binding regions compared to background data sets is shown. The four most statistically significant over-represented motifs in both cases are indicated (**). A consensus binding region based on these preferences is shown as an inset. Upper case letters represent the preferred nucleotides at each position whereas lower case letters represent alternative nucleotides that are favored at particular positions. (**C**) WebLogo representation of the nucleotide frequencies surrounding the GTAAACA core motifs found within the FOXK2 and FOXO3 binding regions in comparison to the Genomic background. Shaded rectangles indicate nucleotides whose frequencies are higher than a random distribution using the entire genome as a background. (**D**) Percentage of indicated binding peaks (±200 bp from the summit) that contain the ATGTAAACAAS motif. (**E**) Numbers of the sequences GTAAACA and ATGTAAACAAS in the human genome (unmasked hg18) and in the regions occupied by FOXK2 or FOXO3.

The large overlap in binding regions for FOXK2 and FOXO3 could be explained by the presence of either two binding motifs allowing co-binding, or mutually exclusive binding of the two factors to the same motif. To distinguish between these possibilities we first determined the distance between the summits of the FOXO3 and FOXK2 binding peaks in the FOXK2/O3 shared category and found that the majority (81%) were within 160 bp, suggesting that they might be occupying the same motif (Supplementary Figure S4A). Indeed, nearly 80% of the FOXO3 peaks in the FOXK2/O3 shared category contained only one FOX binding motif (Supplementary Figure S4B). It is possible that FOXO3 might bind a more degenerate motif than FOXK2 which was not detected by this motif search. However, *de novo* motif searching of FOXK2 and FOXO3 ChIP-seq data sets identified similar over-represented FOX motifs as the top scoring hits (Figure 2A and Supplementary Figure 4C). Moreover, a more targeted approach was used to investigate the frequency of permuted versions of the core FOX motif in the FOXK2 and FOXO3 ChIP-seq data sets. This analysis showed little difference in the sequence preferences for FOXK2 and FOXO3 binding with the overall consensus being ^G^/_a_T^A^/_C_AA^C^/_t_A although FOXO3 binds a significantly higher percentage of regions containing the optimal GTAAACA motif than FOXK2 (*P*-value = 1e^−49^ based on a binomial distribution)(Figure 2B). Next, we examined the regions flanking the core binding motif and, after fixing the central optimal GTAAACA motif, we observed a sequence preference of the two bases upstream and downstream from the core motif for FOXK2 (Figure 2C). In contrast, only a weak preference 2 bases after the optimal GTAAACA core motif was seen for FOXO3 (Figure 2C). The broader FOXK2 binding motif ATGTAAACAS (where S = G or C) was found in a large proportion of the FOXK2-specific peaks (>6%) compared to the FOXO3-specific peaks (∼1%) (Figure 2D). Genome-wide, FOXK2 bound a higher proportion of possible ATGTAAACAAS motifs than the available core GTAAACA motifs but this large difference was not observed for FOXO3 (Figure 2E). When further partitioned into shared and uniquely bound regions, FOXK2-specific regions again showed the highest relative binding frequency of the broader ATGTAAACAAS motif compared to GTAAACA motifs (Supplementary Figure S4D).

**Figure 3.**
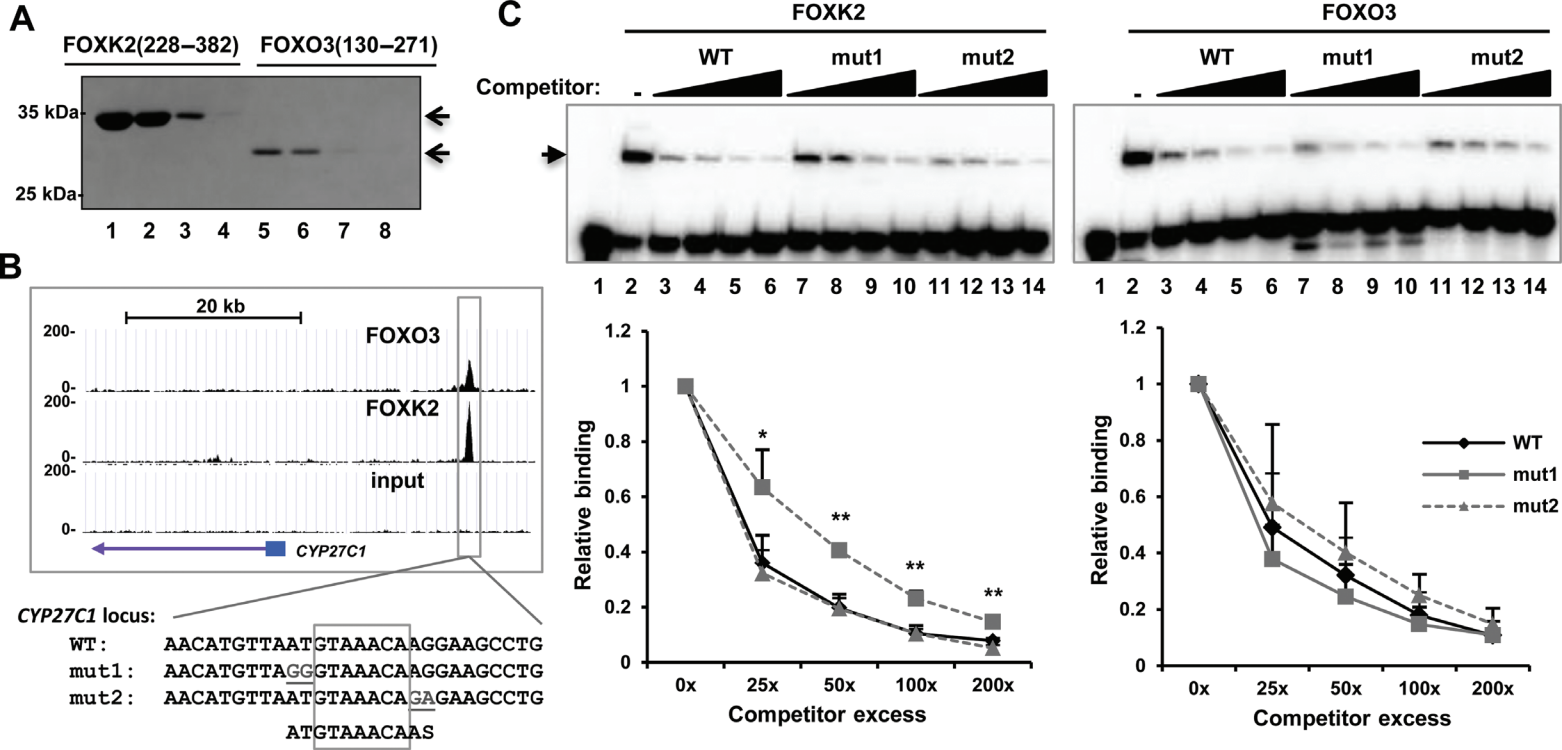
The 5′ flanking region is important for FOXK2 binding to DNA. (**A**) Coomassie staining of SDS–PAGE analysis of 5% of each four separate eluted samples of purified His-tagged FOXK2(228–382) and FOXO3(130–271) (containing the forkhead DNA-binding domain). (**B**) UCSC browser view of the FOXO3 and FOXK2 ChIP-seq peaks of *CYP27C1* locus. DNA sequences of the wild-type and mutant binding regions used in EMSA experiments are shown below. Mutated bases are underlined and the core GTAAACA motif boxed. (**C**) Competition EMSA experiment using increasing concentrations (25x, 50x, 100x and 200x molar excess) of the indicated unlabeled sequences to compete for binding of FOXK2 (left) or FOXO3 (right) proteins to the labelled wild-type (WT) sequence. Protein–DNA complexes are indicated by the arrow. Quantification of the FOX–DNA binding at each concentration of competitor relative to binding in the absence of competitor (taken as 1) is shown below. The error bars represent the standard deviations of three independent experiments. * and ** represent *P* < 0.05 and *P* < 0.01, respectively.

To determine whether the *in vivo* binding preferences of FOXK2 for the core flanking regions are an inherent property of the protein or are influenced by other cellular factors, we purified recombinant proteins encompassing the Forkhead DNA binding domains of FOXK2 and FOXO3 (Figure 3A) and tested their binding *in vitro* to variants of a FOXK2 binding site found associated with the *CYP27C1* locus (Figure 3B). FOXK2 or FOXO3 were incubated with sequences corresponding to the radioactively-labelled wild-type binding site in the presence of increasing concentrations of unlabelled competitors (Figure 3C). FOXK2 binding was competed equally well by the wild-type sequence and a competitor with mutations in the 3′ flanking region (mut2)(Figure 3C, left). In contrast, mutation of the 5′ flanking region (mut1) reduced the effectiveness of FOXK2 binding, and caused reduced competition (Figure 3C, left). On the other hand, all three competitors competed equally well for FOXO3 binding to this site (Figure 3C, right).

Together, these data therefore indicate that FOXK2 and FOXO3 bind to similar core consensus motifs but FOXK2 shows additional sequence preference for sequence flanking this core region, which are especially important in the two 5′ flanking bases.

### FOXK2 and FOXO3 do not compete for DNA binding *in vivo*

**Figure 4.**
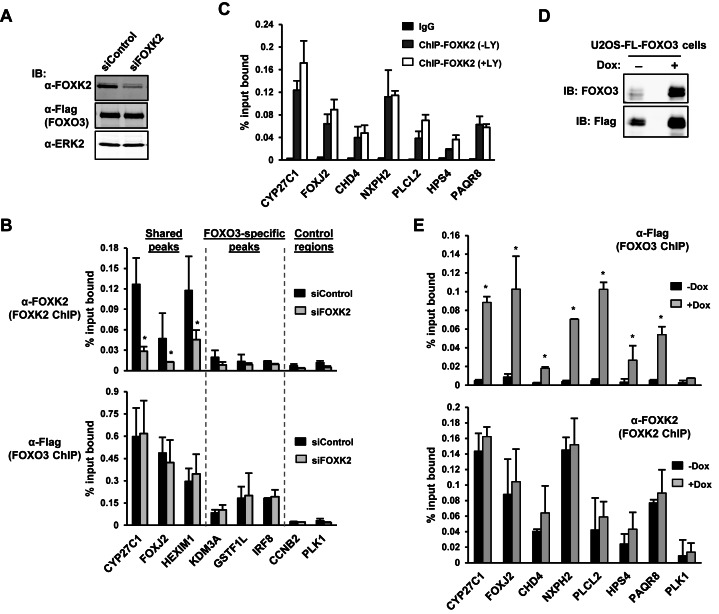
FOXK2 and FOXO3 do not generally compete for binding site occupancy. (**A** and **D**) Western blot analysis showing the level of endogenous FOXK2, FOXO3, and Flag-tagged FOXO3 protein in the U2OS-3xFLAG-FOXO3 cell line following treatment with 1 μg/ml doxycycline and either a non-targeting siRNA (con) or an siRNA targeting FOXK2 (A) or following treatment with or without 1 μg/ml doxycycline (dox) for 24 h (D). Protein expression was determined by immunoblotting (IB) with the indicated antibodies. (B, C and E) ChIP analysis of endogenous FOXK2 or Flag-tagged FOXO3 in U2OS-3xFLAG-FOXO3 (B and E) or U2OS (C) cells. (**B**) Cells were treated with doxycycline for 24 h and then LY294002 for 2 h before crosslinking and also treated with either a non-targeting siRNA (con) or a siRNA targeting FOXK2. ChIP experiments were performed with a FOXK2 (top) or Flag (FOXO3; bottom) antibody on genomic regions associated with the indicated loci. (**C**) ChIP experiments were performed with control IgG or a FOXK2 antibody on genomic regions associated with the indicated loci in cells left untreated or treated with LY294002 for 2 h before crosslinking. (**E**) Cells were treated with doxycycline for 24 h and then LY294002 for 2 h before crosslinking. ChIP experiments were performed with a Flag (FOXO3; top) or FOXK2 (bottom) antibody on genomic regions associated with the indicated loci. The error bars represent the standard deviations from two independent experiments. Where indicated, statistical significance is shown (* = *P*-value < 0.05 with a one tailed t-test (B) or two tailed t-test (E)).

The overlap in binding regions occupied by FOXK2 and FOXO3 and their similar binding sequence preferences, suggest that their binding to a given genomic region should be mutually exclusive. To test this hypothesis, we therefore manipulated the expression levels of FOXK2 and FOXO3 and determined the impact on reciprocal chromatin binding. First, we depleted endogenous FOXK2 (Figure 4A) and determined its impact on FOXO3 binding to a range of FOXK2/FOXO3 shared and FOXO3-specific binding regions. FOXK2 binding was substantially reduced on all of the shared regions (Figure 4B, top). However, no reciprocal increase in FOXO3 binding was observed (Figure 4B, bottom). Similarly, we tested the effect of FOXK2 depletion on FOXO3 occupancy at sites uniquely occupied by FOXK2, and again no increase in FOXO3 binding levels was observed (Supplementary Figure S5). Next, we asked whether increasing the nuclear concentration of FOXO3 by treating cells with LY294002 affected FOXK2 binding to chromatin. However, no decreases in FOXK2 binding were detected under these conditions (Figure 4C) despite the increased binding of FOXO3 (see Supplementary Figure S3A). Finally, we overexpressed FOXO3 (Figure 4D) and asked whether this affected FOXK2 binding to chromatin. However, again, there was no effect of FOXK2 binding despite the large increases in FOXO3 binding observed at these sites (Figure 4E).

Collectively, these data therefore suggest a model whereby FOXK2 and FOXO3 share partial occupancy of the binding regions and this equilibrium is not disturbed by perturbations in the concentrations of either factor.

### FOXJ3 shares an overlapping binding profile with FOXK2 and FOXO3

**Figure 5.**
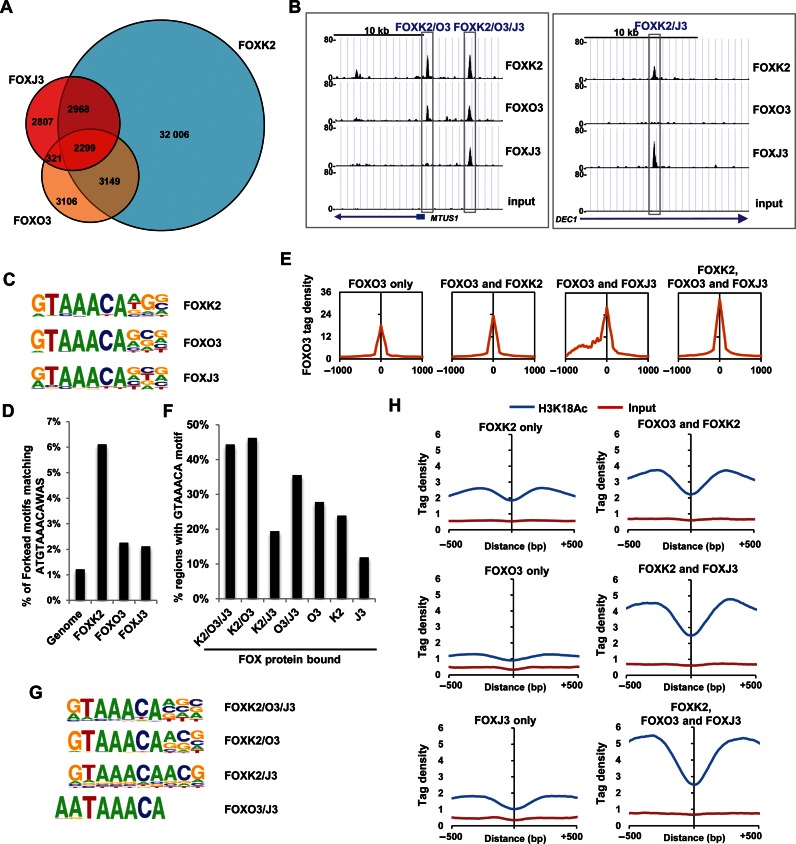
FOXK2, FOXO3 and FOXXJ3 exhibit extensive overlap in chromatin binding. (**A**) Venn diagram showing overlapping binding regions shared between FOXK2, FOXO3 and FOXJ3. If multiple peaks from a single FOX protein overlapped, they were merged to a single event. (**B**) UCSC genome browser views of FOXK2, FOXO3 and FOXJ3 binding profiles associated with the indicated loci illustrating binding regions shared between the indicated FOX proteins. (**C**) WebLogo representation of FOX motifs identified by de novo motif discovery in the FOXK2, FOXO3 and FOXJ3 binding regions. (**D**) Percentage of indicated binding peaks (±200 bp from the summit) that contain the ATGTAAACAAS motif. (**E**) Average tag densities surrounding the summits (±1 kb) of the FOXO3 binding regions, either uniquely or also associated with binding of the indicated additional FOX proteins. (**F**) Percentage of indicated classes of binding regions (±200 bp from the summit) that contain the core GTAAACA motif. (**G**) WebLogo representation of the top ranking FOX motifs identified by de novo motif discovery in regions bound by the indicated combinations of FOXK2, FOXO3 and FOXJ3. (**H**) Average tag densities (counted in 10 bp bins) of H3K18 acetylation surrounding the summits (±500 bp) of the regions associated with binding of the indicated FOX proteins.

In addition to FOXK2 and FOXO3 binding, it is also possible that other FOX transcription factors might exhibit overlapping chromatin binding profiles. This would help explain why no mutually exclusive binding was observed upon manipulating the levels of a single FOX transcription factor. To test this hypothesis, we determined the genome-wide binding profile of an additional FOX protein that is highly expressed in U2OS cells, FOXJ3 (Supplementary Figure S1). Again we used the same experimental system, and inducibly expressed FOXJ3 as a triple Flag-tagged fusion protein in U2OS cells (U2OS-3xFLAG-FOXJ3 cells)(Supplementary Figure S6A). Substantial overlaps were seen between FOXJ3 binding regions and those bound by other FOX proteins. Over 60% (5267) of the FOXJ3 binding regions were also occupied by FOXK2 (Figure 5A). Moreover, there was also substantial overlap (27%) of FOXJ3 binding with sites co-bound by both FOXK2 and FOXO3 (Figure 5A). Interestingly, the majority of the most significantly enriched GO terms were for genes associated with regulatory regions bound by either FOXK2 alone or co-bound by FOXK2, FOXO3 and FOXJ3 (Supplementary Figure S7). In the latter case, this suggests a functional importance and the most significant category was ‘apoptotic signalling pathway’, a process previously attributed to regulation by FOXO transcription factors (13). Different categories of binding regions can therefore be defined bound either by FOXK2 and FOXO3 (FOXK2/O3), by FOXK2 and FOXJ3 (FOXK2/J3) or by all three FOX proteins (FOXK2/O3/J3) (e.g. see Figure 5B), and these categories differ in the biological functions of their target gene repertoires. Again, we asked whether manipulating FOXK2 or FOXJ3 levels might affect reciprocal binding of the other factor but overexpression of FOXJ3 did not reduce FOXK2 binding to chromatin (Supplementary Figure S6B) and reciprocally, depletion of FOXK2 did not affect FOXJ3 binding to a range of shared binding regions (Supplementary Figure S6C and D). These results are therefore consistent with a model in which saturation is achieved by multiple FOX proteins binding to the same regions with partial occupancy.

Next, we examined the properties of each binding region category to identify any defining features that might point to mechanisms for binding specificity generation or any differences in functionality. Similar core FOX motifs were identified when searching for *de novo* motifs in the entire FOXK2, FOXO3 and FOXJ3 data sets (Figure 5C) and the FOXK2 binding regions showed the biggest enrichment for the longer ATGTAAACAAS motif (Figure 5D; Supplementary Figure S8A). This association was also seen when analysing regions bound by combinations of FOX proteins with FOXK2-specific regions containing the highest proportion of the ATGTAAACAAS motif, and the FOXK2-FOXO3 co-bound category also showing significant enrichment of this motif (Supplementary Figure S8B). We then examined tag densities within each binding region category to examine whether there are qualitative differences in the binding intensity of the FOX peaks. Intuitively, if multiple FOX proteins are competing for binding to the same site, reduced binding of each individual FOX protein would be expected at the triply occupied regions. However, in all cases, there was an increase in average tag density when going from uniquely bound regions by a FOX protein, through doubly bound regions to regions bound by all three FOX proteins which showed the highest tag densities (Figure 5E; Supplementary Figure S8C and D). There was also a trend for an increased frequency of occurrence of matches to the core GTAAACA motif sequence as the number of different FOX binding proteins increased (Figure 5F). One clear exception to this trend was the low number of core GTAAACA motifs found in the FOXK2/J3 shared binding regions. Indeed, *de novo* motif searching identified a less stringent FOX binding motif in the FOXK2/J3 binding regions (Figure 5G) and none of the three highest ranking motifs resembled the core FOX motif from the FOXJ3-specific regions (Supplementary Figure S9A). However, there is a FOX-like binding motif returned further down the list (found in 14% of binding sites) that is enriched in the FOXJ3-specific data set suggesting that there are regions specifically bound by FOXJ3 alone and we have validated FOXJ3 binding to several of these regions (>70% positive; Supplementary Figure S10). Interestingly, the FOX motif found in the FOXO3/J3-bound regions differs significantly from the consensus GTAAACA motif, incorporating two A residues preceding the TAAACA sequence (found in 36% of sites; *P*-value = 1 × 10^−30^)(Figure 5G), indicating that FOXJ3 and FOXO3 can preferentially bind to a different site than FOXK2.

Finally, to begin to understand the local chromatin context of the binding regions, we examined whether the histone acetylation mark H3K18ac was preferentially associated with any particular category of FOX binding region. This mark has previously been shown to be associated with active regulatory regions found in promoters and enhancers (21). There are a total of 61 212 H3K18ac regions and 29 512 of them are also bound by at least one FOX transcription factor demonstrating a strong co-association. Importantly, we found preferential enrichment of H3K18ac surrounding sites shared by all three FOX proteins, with the lowest enrichment at regions uniquely occupied by individual FOX proteins (Figure 5H). The relationship between FOX protein binding and the acquisition of H3K18ac is unclear but acetylation levels are clearly higher around FOX binding motifs bound by FOX proteins rather than unbound motifs (Supplementary Figure S11). Furthermore, 18% of the regions containing both H3K18ac and FOX protein binding contain the GTAAACA FOX binding motif, whereas only 11% of H3K18ac containing regions that lack FOX protein binding contain this motif. Collectively our data demonstrate a strong co-association between FOX protein binding and H3K18ac across the genome and in particular demonstrate that regions bound by multiple FOX proteins appear to be associated with regulatory regions of the genome.

Together, these results demonstrate that the regions which are bound by all three FOX proteins have higher FOX protein occupancy, more surrounding H3K18ac and stronger binding motifs suggesting potential functional relevance. Outside from the triply occupied FOXK2/O3/J3 regions, FOXJ3 has less stringent binding requirements either in uniquely bound regions or in regions also shared with FOXK2 but shows a different binding specificity on sites shared with FOXO3.

### Regions bound by multiple FOX proteins are functionally relevant for FOXO3 activity

**Figure 6.**
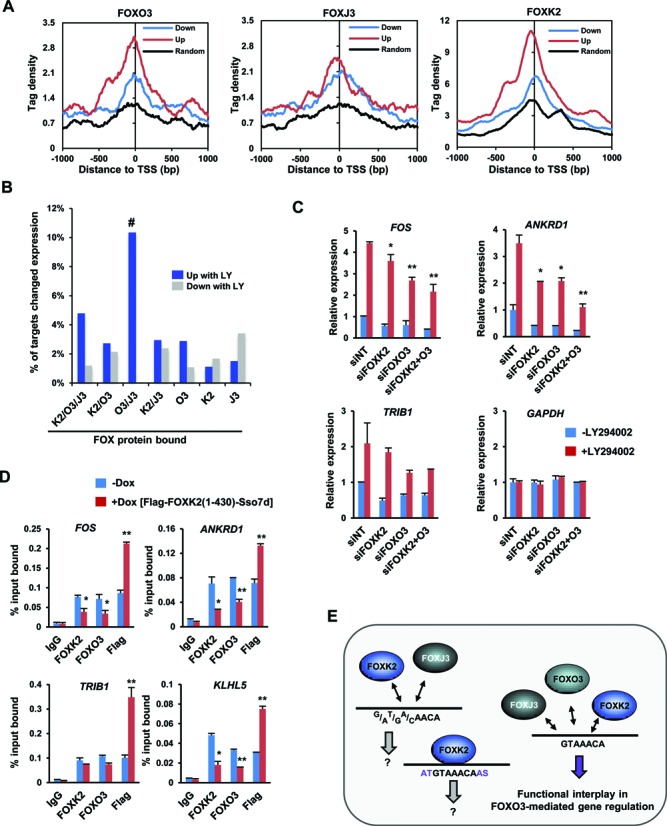
Functional interplay between FOXO3 and FOXK2 at commonly bound regions. (**A**) Average tag densities of ChIP-seq signals from the indicated FOX proteins surrounding the TSS (±1 kb) of genes that are either up- or down-regulated following treatment of U2OS cells with LY294002. A control group of randomly selected genes that are not inducible by LY294002 treatment is shown for comparison. (**B**) Percentage of genes associated with the indicated classes of FOX binding regions that show either up- or down-regulation following LY294002 treatment. (**C**) RT-qPCR analysis of the indicated genes following treatment of U2OS cells with or without LY294002 after pre-treatment with non-targeting (NT) siRNAs, or individually or in combination with siRNAs against FOXK2 or FOXO3. The error bars represent the SEM from three independent experiments. (**D**) ChIP analysis of endogenous FOXK2, FOXO3 or Flag-tagged FOXK2(1–430)-Sso7d in U2OS-3xFLAG-FOXK2(1–430)-Sso7d cells treated with or without doxycycline for 24 h and then LY294002 for 2 h before crosslinking. ChIP experiments were performed on the genomic regions associated with the indicated loci. The error bars represent the SEM from three independent experiments (* = *P*-value < 0.05; ** = *P*-value < 0.01). (**E**) Model depicting specificity determinants and functional outputs from the indicated combinations of FOX protein binding at different subsets of FOXK2 binding regions.

Intuitively, it might be expected that regions uniquely bound by an individual FOX protein would be more likely to confer biological specificity in terms of gene regulatory activities. However, the genomic features associated with the regions occupied by all three FOX proteins suggested potential functional relevance for FOX protein function and this is further underlined by the strong enrichment of GO terms associated with target genes in this category. To distinguish between these two possibilities we focussed on FOXO3 and its target gene network, as the biological functions of the FOXO subfamily and their downstream targets are well characterized (13). We first determined the potential FOXO3 target genes by treating cells with LY294002 to promote FOXO3 nuclear translocation and determined the differentially regulated genes by microarray analysis. In total, 280 genes were up- and 323 genes were down-regulated following LY294002 treatment for 2 h (1.5-fold change, FDR < 0.05; Supplementary Table S2). Next, to associate FOX binding with gene regulatory activities, we examined the tag densities of binding by the different FOX transcription factors in a region spanning 2 kb centred on the transcriptional start sites of genes showing up- or down-regulation. Binding signals for FOXO3 were highest around genes upregulated by FOXO3 (Figure 6A) consistent with it being a direct activator of these genes. However, FOXK2 binding levels were also high around the upregulated genes. We then associated the deregulated genes with FOX binding regions and asked whether any particular category of binding region was associated with FOXO3-mediated upregulation. Genes associated with single FOXK2 or FOXJ3 binding events were more likely downregulated upon LY294002 treatment, whereas those bound by FOXO3 were more likely upregulated. However, LY294002-mediated upregulation was also associated with FOXO3 regions also bound by FOXK2 and FOXJ3 (Figure 6B). These results suggested that genes associated with both FOXO3 and binding of another FOX protein might be more regulated by one or both of these transcription factors. To test this, we focused on the interplay between FOXO3 and FOXK2 and depleted FOXK2, FOXO3 or both and examined the expression of three FOXK2/FOXO3 associated genes following LY294002 treatment. *FOS*, *ANKRD1* and *TRIB1* expression were all reduced following depletion of FOXK2 or FOXO3, with further reductions in *ANKRD1* expression observable upon co-depletion (Figure 6C). This is consistent with a positive role for both FOXK2 and FOXO3 in regulating the expression of these target genes. Previously we were unable to disrupt FOXK2 and FOXO3 binding through reciprocal changes in the concentrations of each active factor to a range of target regions (Figure 4). To further probe this phenomenon, we created a stable cell line that inducibly expresses a fusion of the N-terminal part of FOXK2 (including the FOX DNA binding domain) fused to Sso7d (Supplementary Figure S12A and B). This fusion protein is predicted to bind to DNA in a more stable manner due to the additional binding energy provided by the Sso7d non-specific DNA binding activity (22,23) and hence should compete effectively with FOX proteins exhibiting low residence times on chromatin. *In vitro* binding experiments demonstrated that this is indeed the case as mutations in both the Forkhead domain and Sso7d moiety reduced the DNA binding strength of the FOXK2-Sso7d fusion protein (Supplementary Figure S12C and D). Upon induction of FOXK2-Sso7d expression, binding could be detected at all four loci tested and a concomitant decrease in the binding of both FOXK2 and FOXO3 was observed (Figure 6D). Importantly this was specific to competition for FOX protein binding as no reductions in binding of other transcription factors at the *FOS* promoter was detected (Supplementary Figure S12E). Furthermore, binding of FOXK2-Sso7d caused a significant reduction in the response of *ANKRD1* and *TRIB1* to induction with LY294002, consistent with its ability to disrupt FOXK2 and FOXO3 binding to their regulatory regions (Supplementary Figure S12F).

Collectively, these data point to a model whereby FOXK2 and FOXO3 both act to control the activity of FOXO3-dependent gene expression through regions commonly bound by both factors.

## DISCUSSION

One of the key unanswered questions related to transcriptional control in higher eukaryotes is how functional specificity is achieved for individual members of transcription factor family families when generally, the underlying DNA binding specificity is often highly similar amongst family members (4,24). It is generally assumed that specificity of action is generated through mechanisms that ensure the binding of a unique family member to a given site, either through selective expression in a cell or through other mechanisms such as cooperative binding with other transcription factors as exemplified by members of the ETS transcription factor family (6,25). Here we show extensive overlap in the binding of three FOX transcription factors, FOXK2, FOXO3 and FOXJ3 to the same genomic regions *in vivo*. However, rather than the uniquely bound regions having functional relevance, we demonstrate instead that the regions commonly bound by several FOX transcription factors are more functionally relevant in the context of FOXO3-mediated gene regulation.

Generally, the *in vivo* core DNA binding preferences of FOXK2, FOXO3 and FOXJ3 are very similar and are centred on the GTAAACA motif. However, while FOXK2 and FOXO3 exhibit an overall similar binding specificity with the consensus G/aTA/CAAC/tA, FOXJ3 shows a more relaxed binding specificity with more heterogeneity in the first three bases (Figure 6E). Interestingly, this more degenerate consensus is present in a subset of FOXK2 binding regions that are also bound by FOXJ3, indicating that this sequence determines which FOXK2 binding regions can also act as FOXJ3 binding sites. Furthermore, in regions bound by FOXJ3 and FOXO3, an alternative strong consensus motif AATAAACA is enriched, indicating that in this context, FOXJ3 can bind with a distinct specificity to FOXK2, and helps explain the sub-partitioning of binding events amongst different FOX proteins. Further specificity determinants are apparent in regions bound by FOXK2 and this transcription factor shows additional sequence preferences in the 5′ and 3′ regions flanking the core motif (see Figure 6E). The two A-T base pairs located in the immediate 5′ flanking region appear to be the most important for the FOXK2 intrinsic binding specificity (Figure 3). Previous work indicated that many *in vitro*-derived transcription factor binding motifs show a preference for A-T runs at the 5′ends of the core binding motifs and were hypothesized to help with shape recognition in these flanking regions due to the narrowing of the minor groove in A-T tracts (4). It is possible that the A-T base pairs also play a structural role in aiding FOXK2 binding. To date we have been unable to demonstrate any role for FOXJ3 and FOXK2 DNA specificity determinants in generating functional specificity, other than chromatin binding. Instead the similar intrinsic binding specificities of FOXK2 and FOXO3 for the sequences in their shared sites correlate with functionally important binding events leading to changes in target gene expression.

The overlap in binding of the same regions by FOXK2 and FOXO3 (and often also FOXJ3) provides us with a conundrum. The lack of multiple core FOX binding motifs or different intrinsic specificities for divergent motifs indicates that only one protein can bind directly at a time. However, if that was the case, then manipulating the expression levels of FOXK2 should affect the binding levels of FOXO3 and vice versa. However, this is not observed and suggests a model in which multiple FOX proteins may be in dynamic equilibrium in binding to a given motif leading to ‘partial occupancy’ (Figure 6E). Indeed, it is likely that additional FOX proteins will also bind to the same sites identified here as multiply bound by FOXK2, FOXO3 and FOXJ3, and contribute further to this partial occupancy. Importantly, we were able to disrupt this equilibrium by expressing a FOXK2-Sso7d fusions protein which is anchored more tightly to DNA and hence less able to dissociate rapidly. This fusion protein led to disruptions in target gene expression, demonstrating the importance of the equilibrium binding dynamics for target gene regulation. This behaviour of FOX proteins resembles that seen previously for nuclear hormone receptors GR and ER where partial chromatin occupancy was observed due to their intrinsic low residency times on DNA (26). Thus this phenomenon might have more widespread relevance to other transcription factor families.

Our data indicate that both FOXK2 and FOXO3 are important in target gene expression through their dynamic association with the regulatory regions of FOXO3-regulated genes. It is not clear why both factors are required but one hypothesis could be that FOXK2 is maintaining the local chromatin environment in a suitable state for FOXO3 binding following its activation through the PI3K pathway. This is analogous to the assisted loading mechanism proposed for nuclear hormone receptors (26). Consistent with such a role, FOXK2 has previously been shown to have pioneering activity in promoting AP1 binding to chromatin (15) and is known to recruit chromatin modification complexes to its binding regions (11). Recently, a study suggested that FOXK2 and the closely related FOXK1 functioned antagonistically to FOXO3 in controlling the activity of genes associated with the autophagy pathway (27). Again, both factors apparently bound through the same DNA sites but, it is not clear how FOXK2 can play such apparently opposite roles in modifying FOXO3 function at two different sets of target genes. Finally it is worth noting that others have shown that FOXK2 and FOXO3 can be co-immunoprecipitated, which would allow one protein to be tethered to the DNA while the other is maintained in the local proximity (28). This in part, might explain some of the functional cooperativity seen between FOXK2 and FOXO3.

In conclusion, our studies on FOX transcription factors suggest a model whereby multiple FOX proteins dynamically associate with the same DNA binding regions through the same motif. Each protein may either play a role in establishing/maintaining the correct local environment, may be neutral at a given locus or may impart specific regulatory properties, leading to changes in target gene activity. In the case of FOX proteins, FOXO3 links target gene expression to the signalling response whereas FOXK2 facilitates this activity. It is tempting to speculate that similar mechanisms might also be operative for other families of transcription factors where multiple proteins with overlapping DNA binding specificities exist and would explain how functional specificity can still be achieved despite extensive overlaps in *in vivo* binding profiles.

## ACCESSION NUMBERS

Experimental data are deposited in array express; E-MTAB-3687 (FOXJ3 ChIP-seq), E-MTAB-3695 (histone H3K18 acetylation ChIP-seq), E-MTAB-2204 (FOXK2 ChIP-seq), and E-MTAB-2701 (FOXO3 ChIP-seq) and E-MTAB-3763 (expression microarray data).

## Supplementary Material

SUPPLEMENTARY DATA
