# Supplementary material for: Genome-wide binding studies reveal DNA binding specificity mechanisms and functional interplay amongst Forkhead transcription factors
Source: Nucleic Acids Res. 2015 Nov 17;44(4):1566–78. doi: 10.1093/nar/gkv1120 (PMC4770209; doi:10.1093/nar/gkv1120)
Supplement: SUPPLEMENTARY DATA [file supp_44_4_1566__index.html]

Genome-wide binding studies reveal DNA binding specificity mechanisms and functional interplay amongst Forkhead transcription factors — SUPPLEMENTARY DATA 

# Genome-wide binding studies reveal DNA binding specificity mechanisms and functional interplay amongst Forkhead transcription factors

## SUPPLEMENTARY DATA

- SUPPLEMENTARY DATA
- SUPPLEMENTARY DATA
