# Supplementary material for: Genome-wide binding studies reveal DNA binding specificity mechanisms and functional interplay amongst Forkhead transcription factors
Source: Nucleic Acids Res. 2015 Nov 17;44(4):1566–78. doi: 10.1093/nar/gkv1120 (PMC4770209; doi:10.1093/nar/gkv1120)
Supplement: SUPPLEMENTARY DATA [file supp_gkv1120_nar-02024-f-2015-File004.pdf]

Supplementary Information- Chen et al.

Supplementary Materials and Methods

Plasmid constructs

The wild-type or DNA-binding mutants of FOXK2(1-430) expression vector pAS4318 [pCDNA3-FOXK2(1-430)] and pAS4319 [pCDNA3-FOXK2(1-430)H308A] were constructed by inserting PCR products using ADS1305/ADS4689-pAS4314 or ADS1305/ADS4689-pAS4315 primer pair-templates into pCDNA3 through BamHI and XbaI sites. DNA-binding mutants of FOXK2-Sso7d fusion protein expression vectors in pCDNA5-FRT-TO-3xFLAG were made by Quikchange mutagenesis using the primer-template combinations ADS4640/ADS4641-pAS4314 for pAS4315 [pCDNA5-FRT-TO-3xFLAG-FOXK2(1-430)(H308A)-Ssod7], ADS4642/ADS4643-pAS4314 for pAS4316 [pCDNA5-FRT-TO-3xFLAG-FOXK2(1-430)-Sso7d(W24A)], ADS4640/ADS4641-pAS4316 for pAS4317 [pCDNA5-FRT-TO-3xFLAG-FOXK2(1-430)(H308A)-Sso7d(W24A)], respectively. The inserts from these vectors were further cloned into pCDNA3 which were then used as templates for *in vitro* translation, These vectors were constructed by inserting two fragments into BamHI and NotI sites in pCDNA3, one from digestion of pAS4314 with BamHI and EcoRI and a second by digesting the following plasmids with EcoRI and NotI; pAS4314 for pAS4320 [pCDNA3-FOXK2(1-430)-Sso7d(E35L)], pAS4315 for pAS4321 [pCDNA3-FOXK2(1-430)(H308A)-Sso7d(E35L)], pAS4316 for pAS4322 [pCDNA3-FOXK2(1-430)-Sso7d(E35L)(W24A)] or pAS4317 for pAS4323 [pCDNA3-FOXK2(1-430)(H308A)-Sso7d(E35L)(W24A)] respectively.

Avidin–biotin coupled DNA-binding (ABCD) assay

The *in vitro* biotinylated DNA-binding assays were performed essentially as described previously (Freddie et al., 2007). TnT® T7-coupled transcription/translation system (Promega) was used to express [^{35}S] methionine-labeled proteins *in vitro.* The biotin-linked wild-type or mutant *ANKRD1* probes containing FOX binding sites were created by annealing the oligonucleotides ADS4757 (5'-Biotag-CTATTCCCTGGGTAAACAGCCTGAGGGGAAG-3') / ADS4758 (5'-CTTCCCCTCAGGCTGTTTACCCAGGGAATAG-3')(WT) and ADS4759 (5'-Biotag-CTATTCCCTGGGTAAAAAGCCTGAGGGGAAG-3') / ADS4760 (5'-CTTCCCCTCAGGCTTTTTACCCAGGGAATAG-3') (mut) respectively. *In vitro* translated proteins were pre-cleared using Dynabead M-280 streptavidin (Life Technologies 11206D) before incubating with 1 µg annealed double-stranded biotinylated probes in binding buffer [50 mM HEPES·KOH, pH 7.9, 150 mM NaCl, 0.5% Triton X-100, 2 mM EDTA, 10 µg/ml poly(dI-dC) and protease inhibitors] at room temperature for 30 min. Then 40 µl streptavidin Dynabeads were added to the reaction and incubated for further 30 min. The beads were washed four times with the binding buffer, and precipitated proteins were separated by SDS-PAGE and analysed by Phosphoimaging.

Supplementary references

Freddie CT, Ji Z, Marais A, and Sharrocks AD (2007) Functional interactions between the Forkhead transcription factor FOXK1 and the MADS-box protein SRF. Nucleic Acids Res. 35:5203-12.

Supplementary Figure legends

Supplementary Fig. S1. Expression levels of FOX transcription factors in U2OS cells. The expression values of individual FOX transcription factors in RNAseq data from U2OS cells are shown as FPKM values. Only FOX proteins with FPKM values more than 10 are labelled (data taken from the Human Protein Atlas).

Supplementary Fig. S2. Functionality of the Flag-Tagged FOXO3 protein. (A) Western blot analysis of endogenous and Flag-tagged FOXO3 in U2OS-FOXO3-HF cells treated with the indicated concentrations of doxycycline (Dox). Immunoblotting (IB) was performed with FOXO3, Flag and LMNB1 antibodies. The concentration of Dox used in subsequent experiments is boxed. (B) ChIP analysis of Flag-tagged FOXO3 binding using an anti-Flag antibody to loci associated with the indicated FOXO3 target genes and the negative control region located in the *PLK1* promoter. Parental IP represents ChIP analysis using anti-Flag antibody in U2OS T-REX cells. Where indicated, statistical significance is shown (*=P-value< 0.05 with a two tailed t-test). (C) Immunofluorescence analysis of endogenous FOXO3 in parental U2OS T-REX cells (left; green) or Flag-tagged FOXO3 in U2OS-FOXO3-HF cells (right; red). Nuclei were co-stained with DAPI (blue). Cells were either grown in FCS alone or in FCS with LY294002 added for 2 hrs.

Supplementary Fig. S3. Characterisation of FOXO3 binding regions. (A) ChIP-qPCR analysis of Flag-tagged FOXO3 binding (using an anti-Flag antibody) to chromatin regions associated with the indicated genes. Data are presented as % input bound and also fold enrichment over a control ChIP experiment performed on the parental U2OS T-REX host

cells (indicated above each bar). Error bars represent the standard deviation from 3 independent experiments. Where indicated, statistical significance is shown (*=P-value< 0.05 with a two tailed t-test). Regions associated with the *CCNB1* and *PLK1* promoters are used as negative controls. (B) Top ten gene ontology terms corresponding to the "biological process" category for the genes associated with FOXO3 binding regions.

Supplementary Fig. S4. **Sequence characteristics of FOXO3 binding regions.** (A) Distribution of summit to summit distances of regions bound by FOXK2 and FOXO3. Data were binned in 20 bp intervals and the % of regions in each bin shown. The shaded area shows the total number of regions showing summit-summit distances of <160bp. (B) Frequency of occurrence of the Forkhead motif RTMAAYA in binding regions showing binding of both FOXK2 and FOXO3. (C) WebLogo representation of over-represented motifs identified by *de novo* motif discovery in the FOXO3 (left) and FOXK2 (right) binding regions. Rank orders are shown according to binomial P-values. (D) Numbers of the sequences GTAAACA and ATGTAAACAAS in the human genome (unmasked hg18) and in the regions occupied by FOXK2 alone, FOXO3 alone or by both FOXK2 and FOXO3.

Supplementary Fig. S5. The interplay between FOXK2 and FOXO3 binding to chromatin. ChIP analysis of endogenous FOXK2 (A) or Flag-tagged FOXO3 (B) in U2OS-3xFLAG-FOXO3 cells. Cells were transfected with either a non-targeting siRNA (NT; black bars) or a siRNA targeting FOXK2 (grey bars), followed by treatment with doxycycline for 24 hrs and then LY294002 for 2 hours before crosslinking. ChIP experiments were performed with a FOXK2 (A) or Flag (FOXO3; B) antibody on genomic regions associated with the indicated loci. The error bars represent the standard deviations from two independent experiments. (*=P-value< 0.05, **=P-value< 0.01 with a one tailed t-test).

Supplementary Fig. S6. **The interplay between FOXK2 and FOXJ3 binding to chromatin.** (A) Western blot analysis of endogenous and Flag-tagged FOXJ3 in U2OS-FOXJ3-HF cells treated with the indicated concentrations of doxycycline (Dox). Immunoblotting (IB) was performed with FOXJ3 and Flag antibodies. The parental U2OS T-REX cell line is shown as a control (lane 1). (B and D) ChIP analysis of endogenous FOXK2 or Flag-tagged FOXJ3 in U2OS-FL-FOXJ3 cells. (B) Cells were left untreated or were treated with doxycycline for 24 hrs. ChIP experiments were performed with a Flag (FOXJ3) or FOXK2 antibody on genomic regions associated with the indicated loci. The error bars represent the standard deviations from two independent experiments. (D) Cells were treated with either a non-targeting siRNA (siControl) or a siRNA targeting FOXK2 for 24 hrs before treating with doxycycline for another 24 hrs. ChIP experiments were performed with a FOXK2 or Flag (FOXO3) antibody on genomic regions associated with the indicated loci (*=P-value< 0.05; **=P-value< 0.01). (C) Western blot analysis showing the level of endogenous FOXK2, endogenous FOXO3 and Flag-tagged FOXJ3 protein in the U2OS-FL-FOXJ3 cell line following treatment with 1 μg/ml doxycycline and either a non-targeting siRNA (con) or an siRNA targeting FOXK2.

Supplementary Fig. S7. **Different categories of FOX target genes are associated with distinct biological processes.** Top ten gene ontology terms corresponding to the "biological process" category for the genes associated with the binding regions from the indicated combinations of FOX transcription factors. Terms associated with apoptosis and autophagy are boxed.

Supplementary Fig. S8. **Sequence and binding characteristics of FOX protein binding regions.** (A and B) Frequency of the sequences GTAAACA and WWGTAAACAWS in the human genome (unmasked hg18) and within the regions (±200 bp from the summit) occupied by FOXK2, FOXO3 or FOXJ3 (A) or in regions partitioned according to occupancy by either FOX protein alone or in combination with other FOX proteins (B). The percentages of all bound GTAAACA motifs corresponding to the extended WWGTAAACAWS motif and associated P-values are shown. (C and D) Average tag densities surrounding the summits (±1 kb) of the FOXK2 and FOXJ3 binding regions, either uniquely or also associated with binding of the indicated additional FOX proteins.

Supplementary Fig. S9. **Over-represented sequence motifs in FOX protein binding regions.** WebLogo representation of the three top ranked motifs identified by *de novo* motif discovery in regions bound by the indicated combinations of FOXK2, FOXO3 and FOXJ3. Boxed motifs correspond to Forkhead-like binding sites.

Supplementary Fig. S10. Validation of FOXJ3 binding to chromatin. ChIP-qPCR analysis of Flag-tagged FOXJ3 binding (using an anti-Flag antibody) to chromatin regions associated with the indicated genes. Genes were selected based on binding uniquely to FOXJ3 and not FOXO3 and FOXK2 in ChIP-seq experiments. Data are presented as % input bound in cells treated with doxycycline (doxy) for 24 hrs and also fold enrichment over a control (C) ChIP experiment performed in the un-induced U2OS-3Flag-FOXJ3 cells (indicated above each bar). *Mcm3int9* represents a negative control region. Error bars represent the standard deviation from three independent experiments. (*=P-value< 0.05, **=P-value< 0.01).

Supplementary Fig. S11. FOX protein bound FOX binding motifs are associated with H3K18 acetylation. Average tag densities (counted in 10 bp bins) of H3K18 acetylation surrounding the summits (±1500 bp) of the regions associated with binding of either FOXK2, FOXO3 or FOXJ3 proteins that either contain (blue line) or lack (red line) the GTAAACA FOX binding motif.

Supplementary Fig. S12. Characterisation of the FOXK2-Sso7d fusion protein. (A) Schematic diagram of the FOXK2-Sso7d fusion protein constructs. The locations of the FHA and FOX DNA binding domains are shown and red crosses indicate the locations of the indicated point mutations. Note that SSod7 contains an E35L mutation which inactivates its RNAase activity and where indicated, a W24A mutation which abolishes its DNA binding activity. (B) Western blot analysis of endogenous FOXK2, FOXO3 and Flag-tagged FOXK2(1-430)-Sso7d fusion protein in U2OS-FOXK2(1-430)-Sso7d-HF cells in the presence and absence of doxycycline (Dox). Immunoblotting (IB) was performed with FOXK2, FOXO3 and Flag antibodies and ERK2 was detected as a loading control. (C and D) DNA binding was assessed by a biotin-streptavidin based pulldown assay using the indicated ^{35}S-labelled *in vitro* translated proteins and biotinylated oligonucleotides containing the wild-type or a mutated version of the Forkhead DNA binding motif located in the FOXK2 binding region associated with the *ANKRD1* locus. Quantification of three independent experiments is shown and error bars represent standard deviation. Addition of the Sso7d moiety increases the binding of FOXK2 to DNA (C, compare lanes 2 and 4). Mutations of either the FOXK2 or Sso7d binding residues reduces the binding of the fusion protein (D), demonstrating their independent contributions to DNA binding. (E) ChIP analysis of endogenous FOXK2, FOXO3, ELK1, SRF or Flag-tagged FOXK2(1-430)-Sso7d in U2OS-3xFLAG-FOXK2(1-

430)-Sso7d cells treated with or without doxycycline for 24 hrs. ChIP experiments were performed on the genomic regions associated with the *TRIB1* locus or the control *FOS* proximal promoter region. The error bars represent the standard deviation from three independent experiments. (F) RT-qPCR analysis of the indicated genes following treatment of U2OS-3xFLAG-FOXK2(1-430)-Sso7d cells with or without LY294002 and with or without doxycycline (Dox) treatment to induce FOXK2(1-430)-Sso7d expression. Data are shown relative to maximal expression of each gene (taken as 1). The error bars represent the standard deviation from three independent experiments. *= P-value<0.05 and **= P-value<0.01.

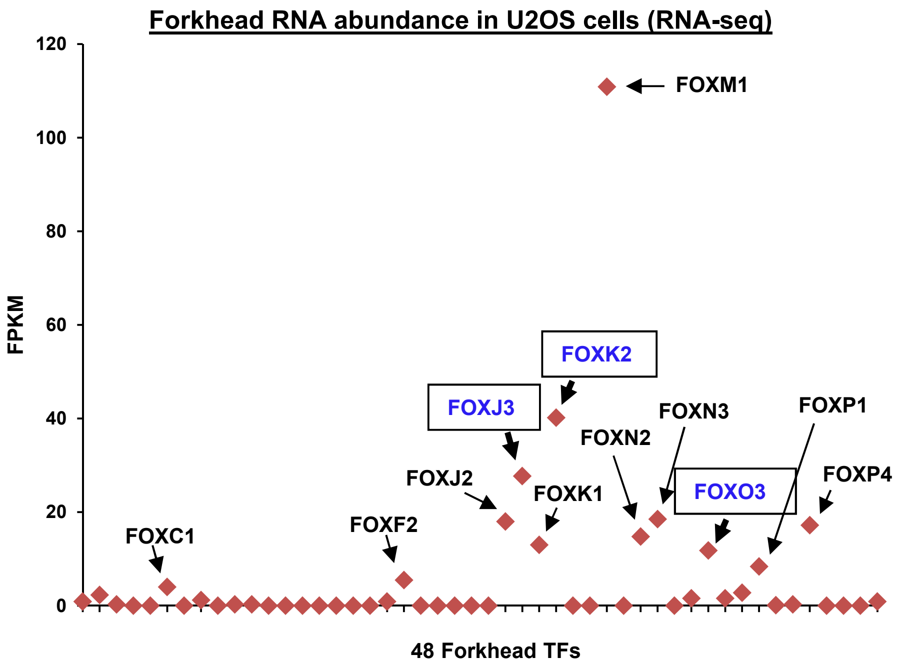

Supplementary Fig. S1 Chen et al., 2015

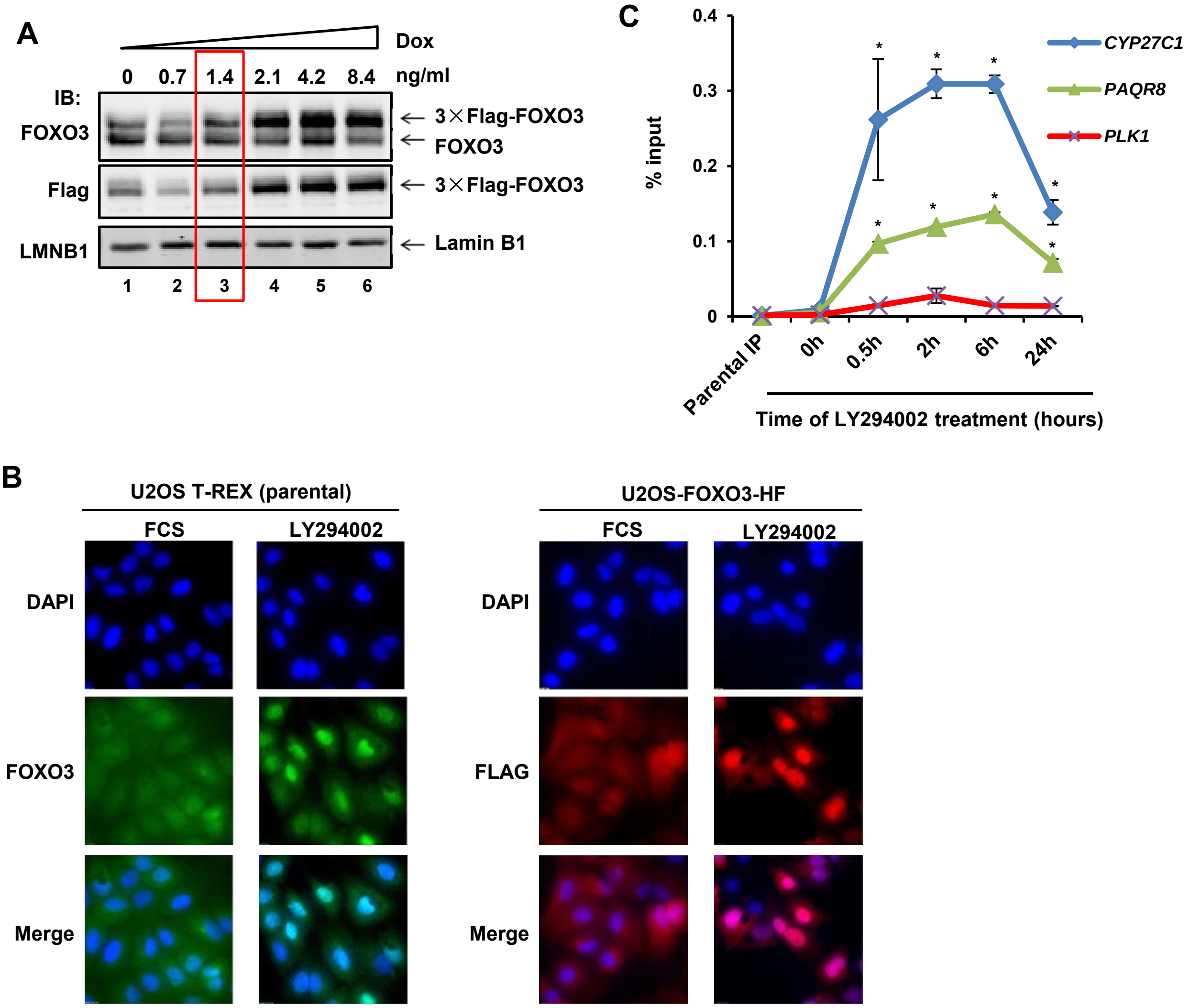

Supplementary Fig. S2 Chen et al., 2015

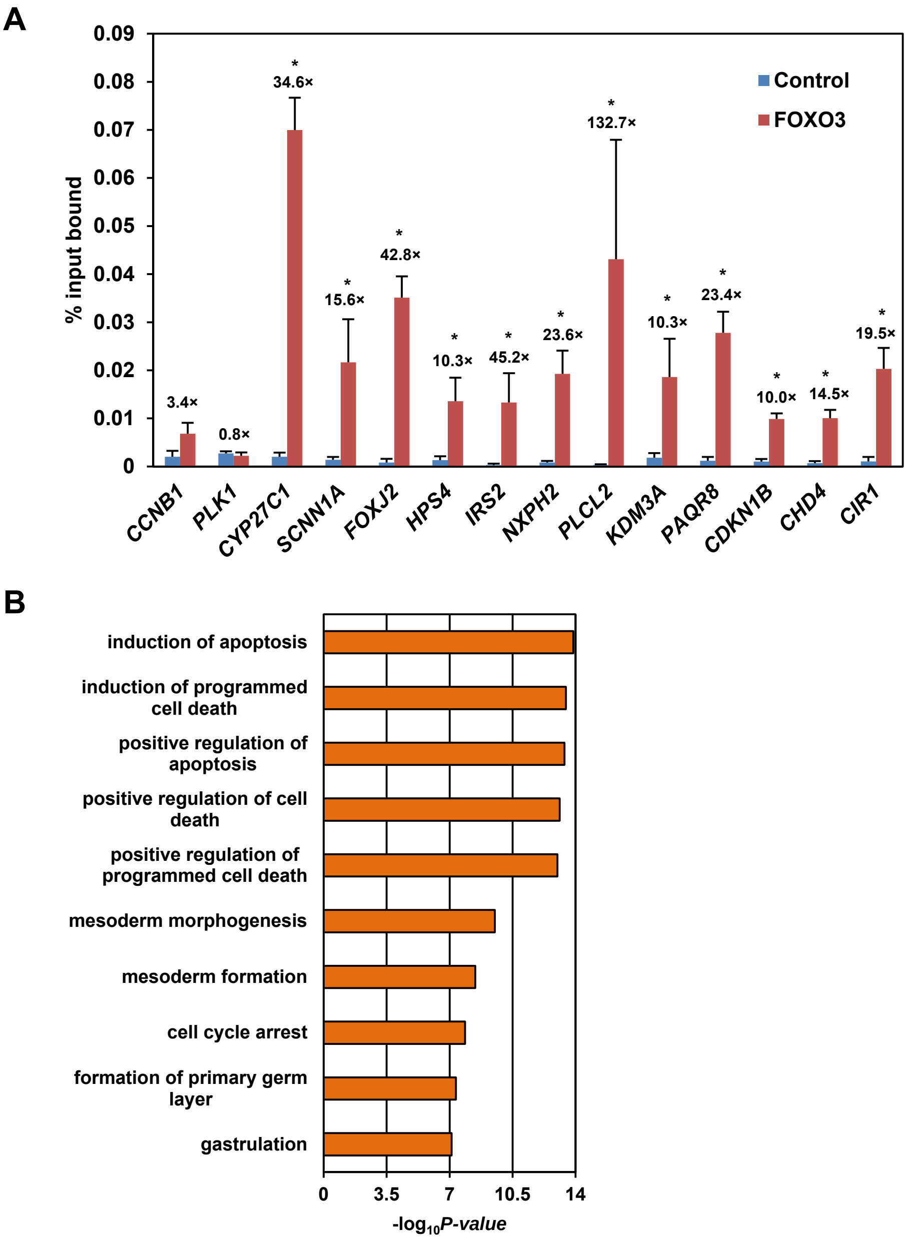

Supplementary Fig. S3 Chen et al., 2015

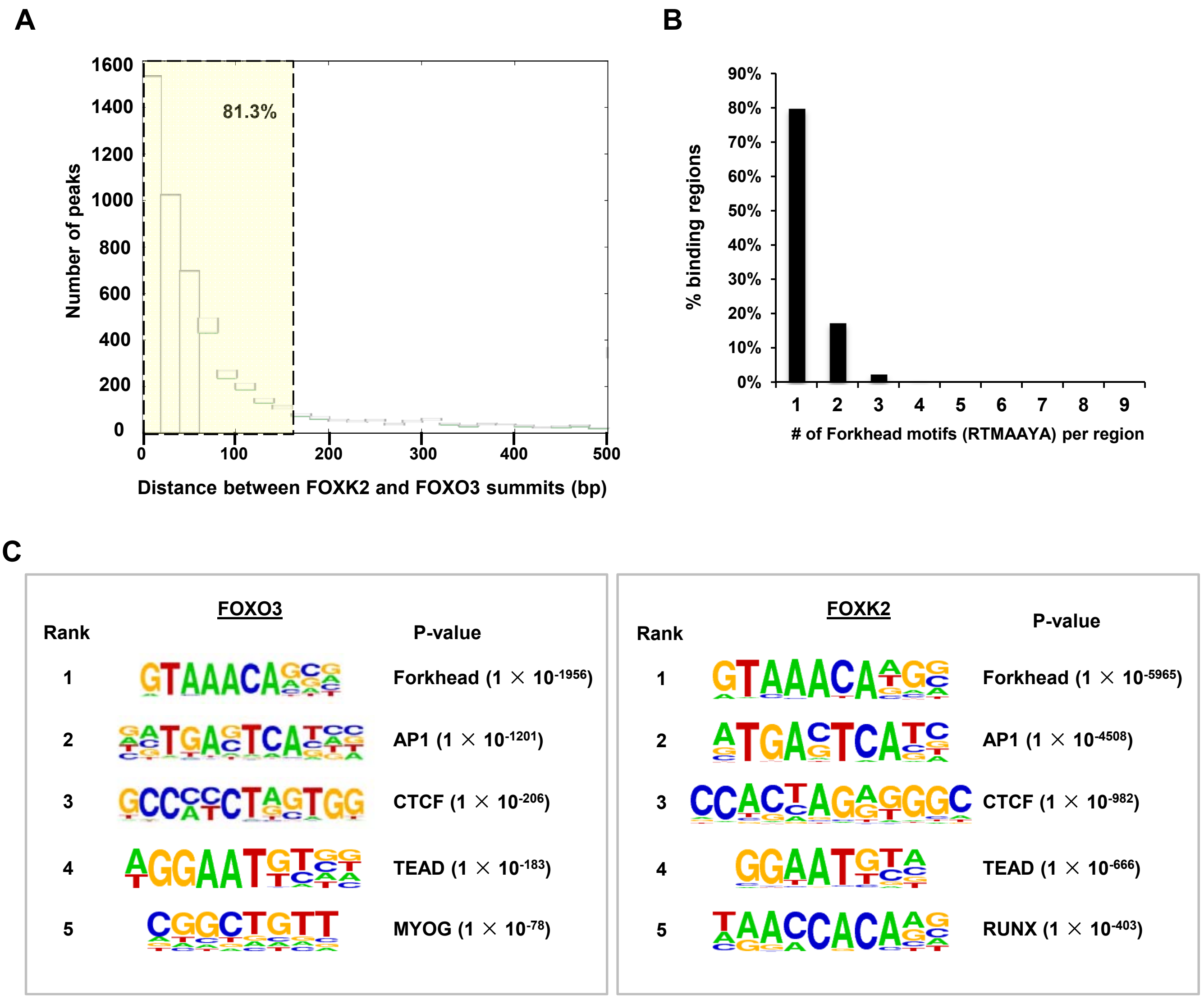

D

Motif	# in genome (hg18)	# (%) occupied by FOXK2 specific	# (%) occupied by shared peaks	# (%) occupied by FOXO3 specific
GTAAACA	472,221	6,017 (1.3%)	1,071 (0.2%)	787 (0.2%)
ATGTAAACAAS	5,736	364 (6.3%)	28 (0.49%)	10 (0.2%)

Supplementary Fig. S4 Chen et al., 2015

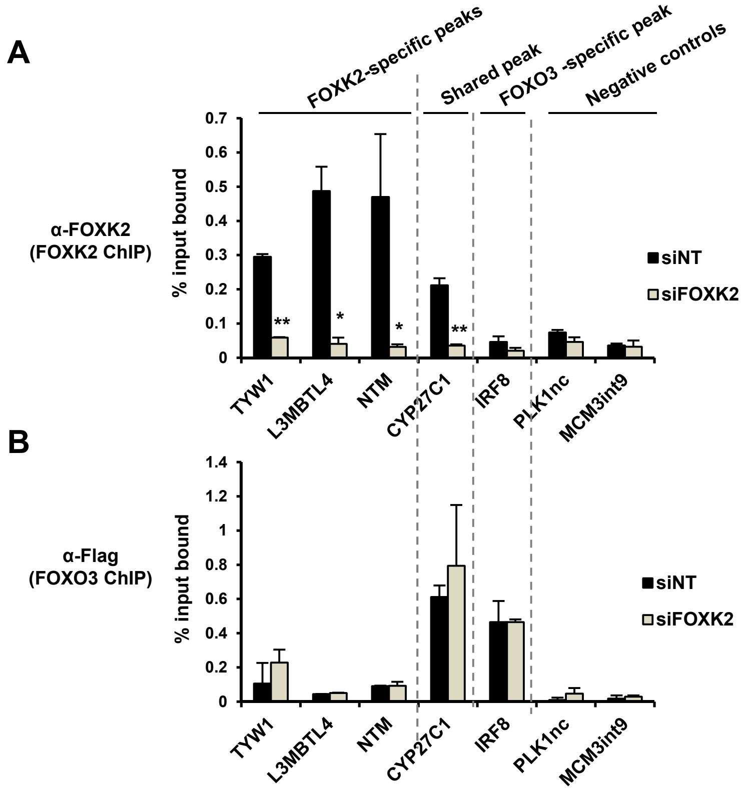

Supplementary Fig. S5 Chen et al., 2015

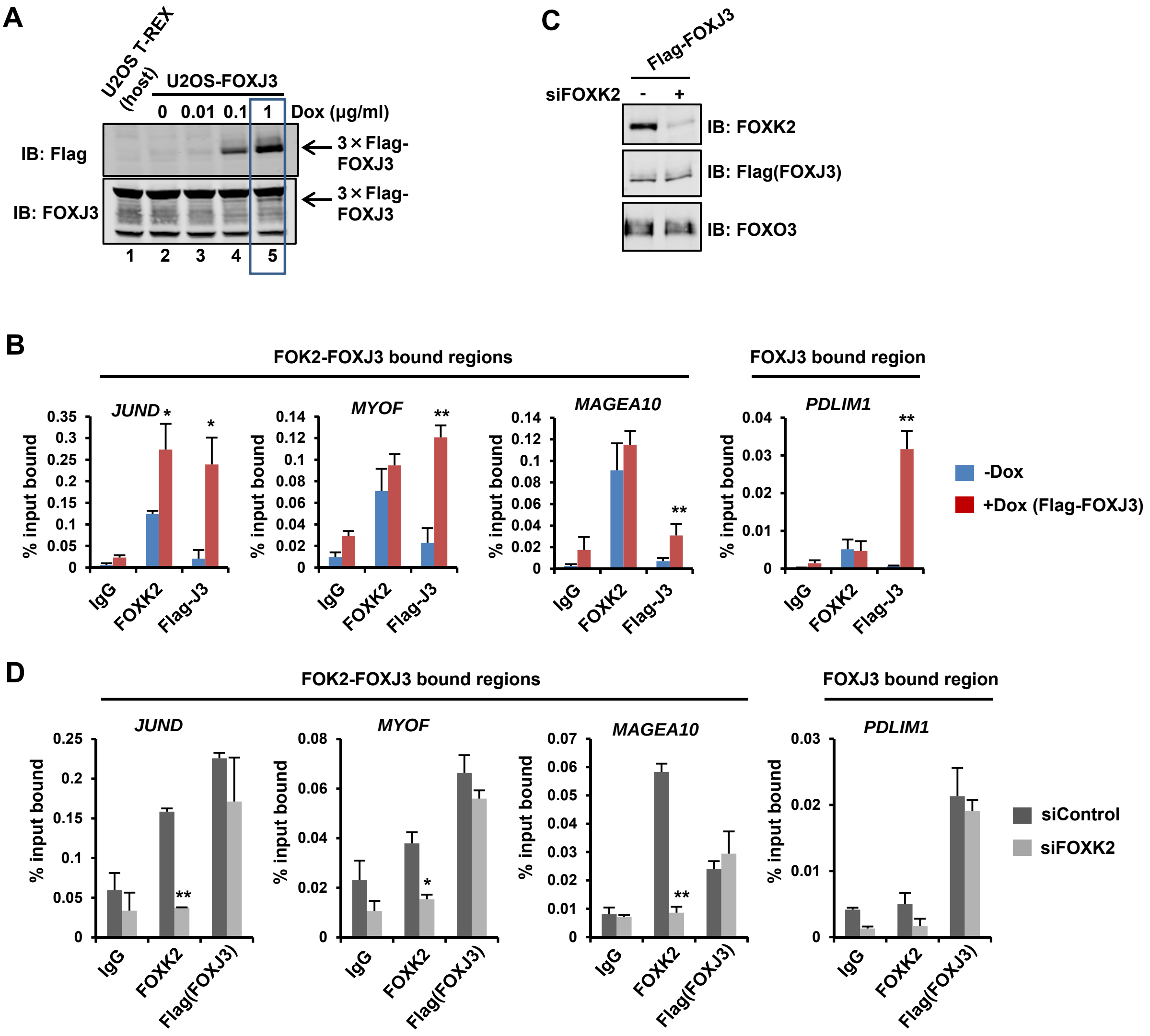

Supplementary Fig. S6 Chen et al., 2015

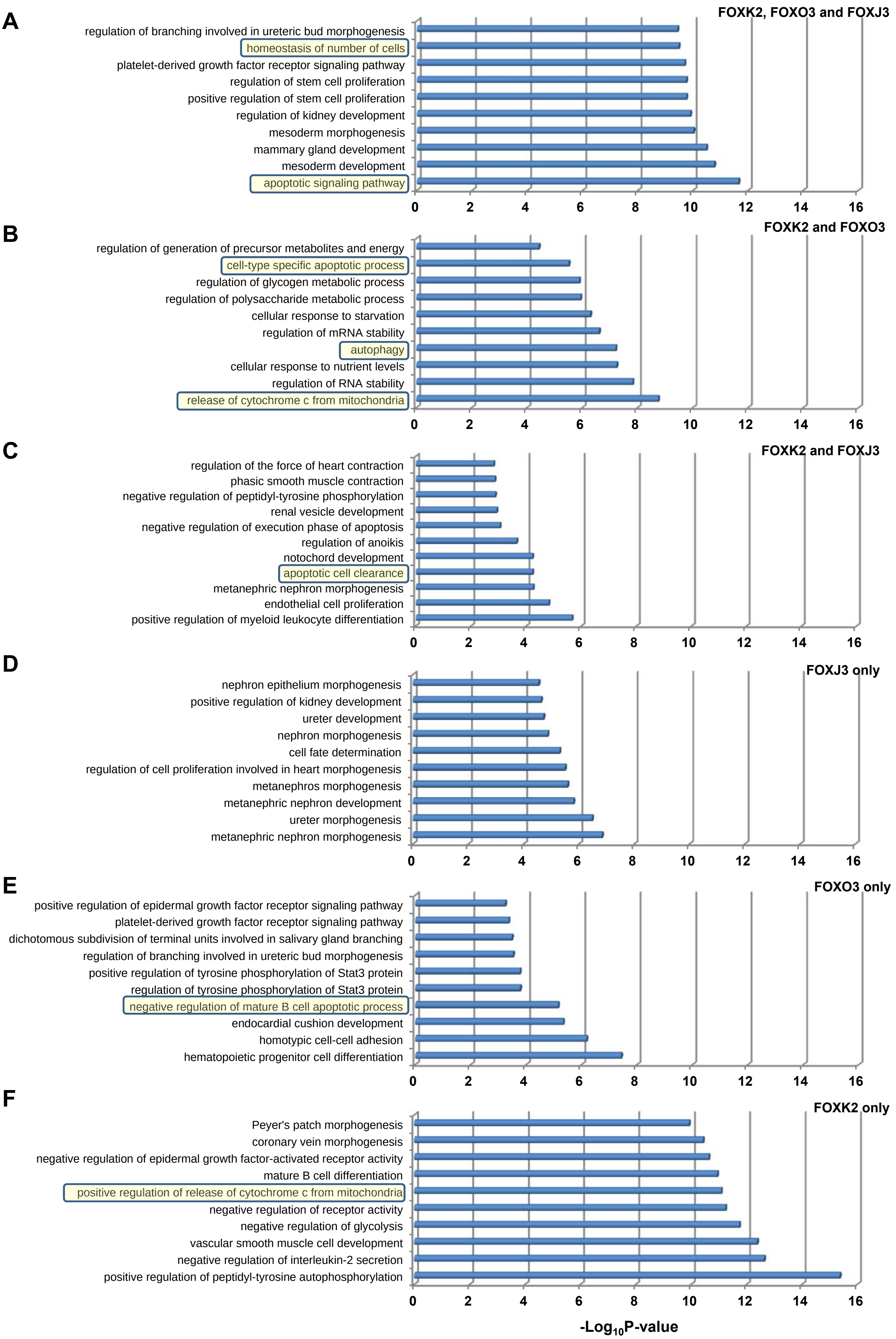

Supplementary Fig. S7 Chen et al., 2015

A

	hg18_genome	FOXJ3	FOXK2	FOXO3
GTAAACA	472221	1088	7713	2703
ATGTAAACAAS	5736	21	471	61
# ATGTAAACAAS / # GTAAACA	1.21%	1.93%	6.11%	2.26%
P-value (hypergeometric)	N/A	0.012	1.08e-178	2.77e-06

B

	hg18_genome	FOXJ3-specific	FOXK2-specific	FOXO3-specific	FOXK2-FOXJ3	FOXK2-FOXO3	FOXO3-FOXJ3	FOXK2-FOXO3-FOXJ3
GTAAACA	472221	167	5749	699	152	651	62	416
ATGTAAACAAS	5736	5	359	9	4	21	1	6
# ATGTAAACAAS / # GTAAACA	1.21%	2.99%	6.24%	1.29%	2.63%	3.23%	1.61%	1.44%
P-value (hypergeometric)	N/A	0.037	1.32e-138	0.13	0.076	4.56e-05	0.36	0.15

C

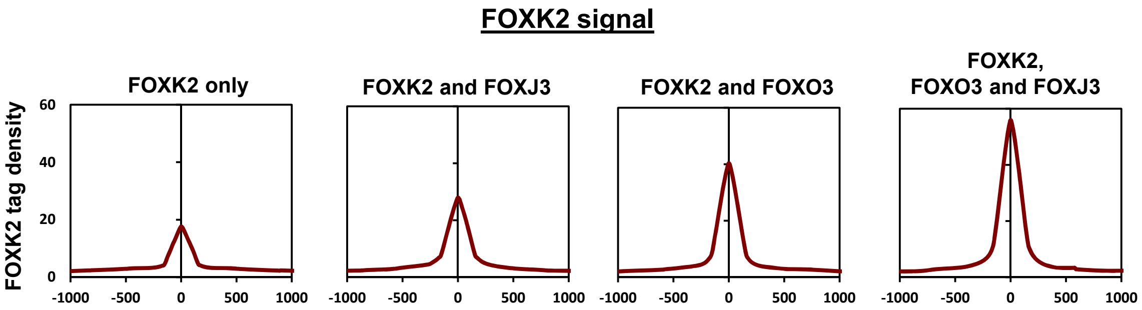

D

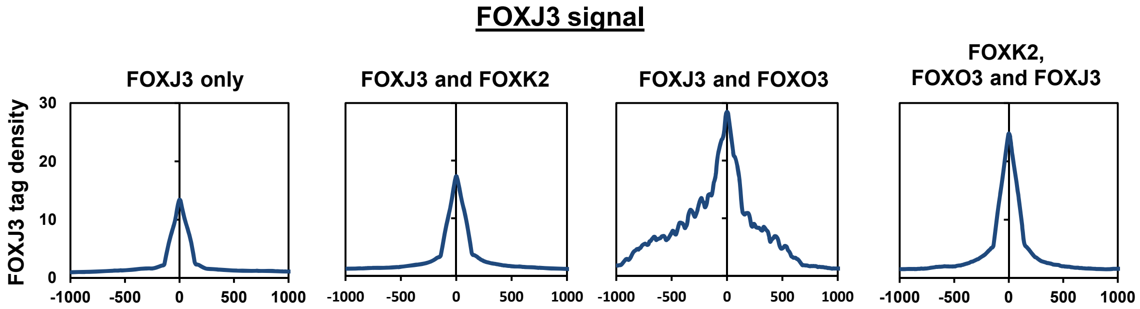

Supplementary Fig. S8 Chen et al., 2015

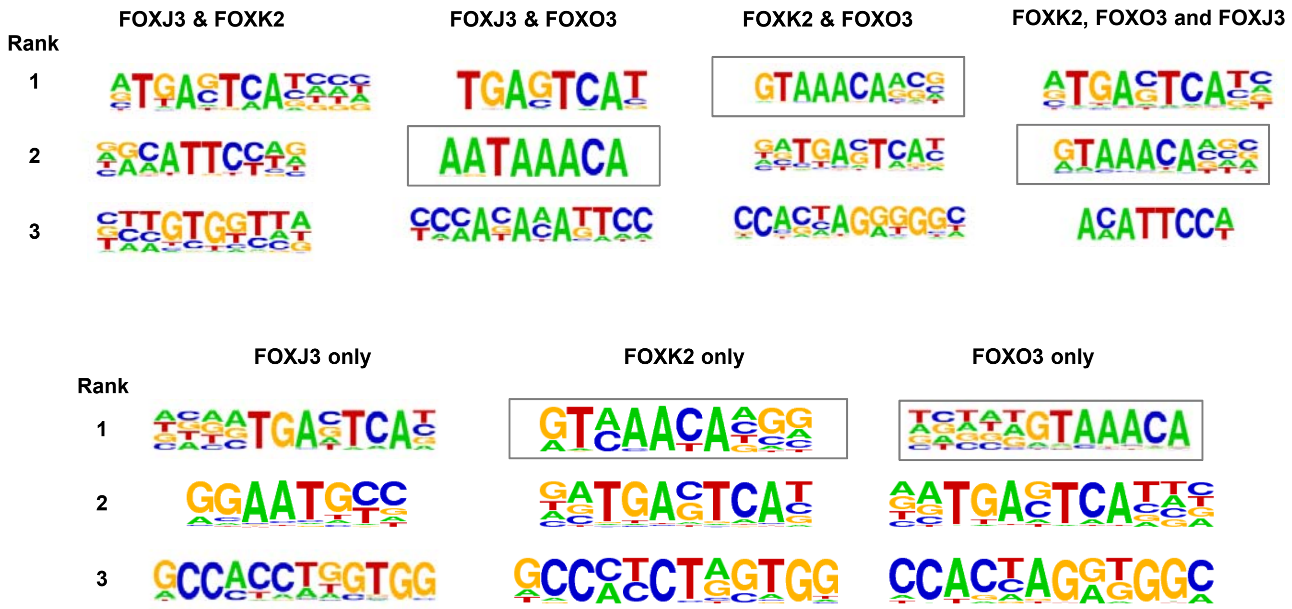

Supplementary Fig. S9 Chen et al., 2015

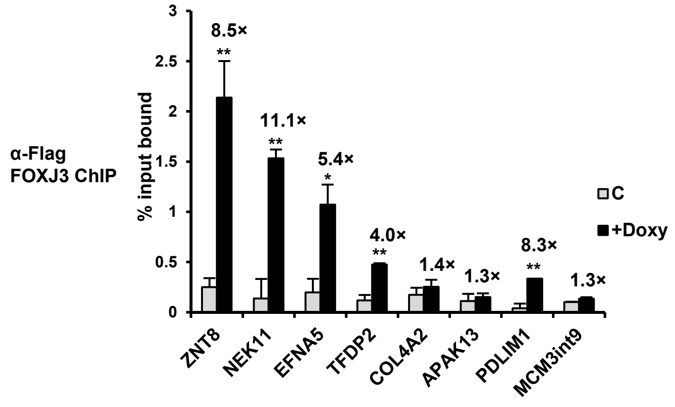

Supplementary Fig. S10 Chen et al., 2015

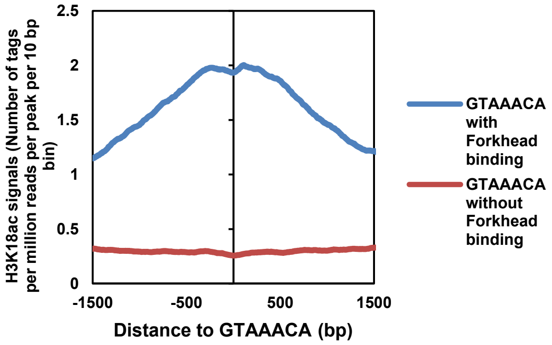

Supplementary Fig. S11 Chen et al., 2015

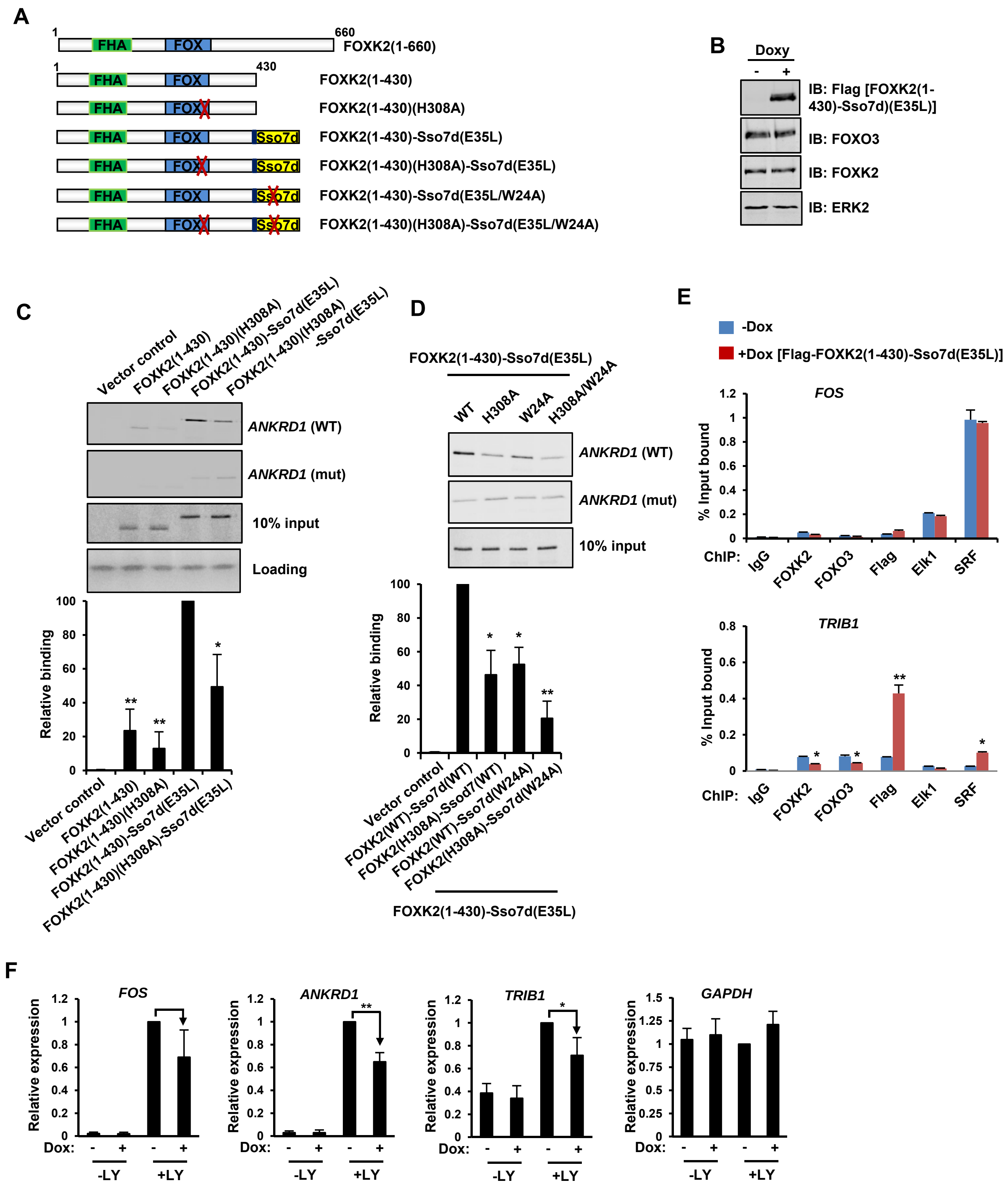

Supplementary Fig. S12 Chen et al., 2015

ASSOCIATED GENE	PRIMER NUMBER	SEQUENCE	REGION AMPLIFIED (Mar. 2006 (hg18) assembly)	PCR PRODUCT SIZE (bp)
CYP27C1	ADS2772<br>ADS2773	GCCTGTTTCCTCCCTGTAGA<br>CCCAGCCCTCAAGATGTTT	chr2:127700750+127700911	162
PLK1	ADS2808<br>ADS2809	CCCCTGGTAGGAGGTCTGTT<br>AAGCGGAAGCTAACCCAAAT	chr16:23594785+23594967	183
CCNB2	ADS2824<br>ADS2825	TGCGAGAGTGCATCTTGTGT<br>CGCCGTTAGGACTGCTCTC	chr15:57184527+57184680	154
PAQR8	ADS2928<br>ADS2929	TGGGAACCCAAAGTAAAGGA<br>ATGGGTGCAACTCCTGCTTA	chr6:52325337+52325478	142
FOXJ2	ADS2941<br>ADS2942	TCCTTTCCTCCGTGTCATTC<br>CGTTGCCTTCAAACAAGGTC	chr12:8070132+8070314	183
GTSF1L	ADS2949<br>ADS2950	GGTGTTGGTGGGAGTGTCTT<br>AGAGCGGCTGATAAACAGGA	chr20:41844265+41844413	149
IRF8	ADS2953<br>ADS2954	AGGCATCTTTGCCATGAGTC<br>AAGGATTTGTCGACCGTCTG	chr16:84514888+84515056	169
HEXIM1	ADS2957<br>ADS2958	AACCCGCCTCTTCGTCTT<br>GAAGGATATCGCGAGCACAT	chr17:40580873+40581022	150
JUND	ADS4645<br>ADS4646	CCGTAGAAGGGTGTTTCCAT<br>GCTATAAGAGGGCGCACAAG	chr19:18253275+18253463	188
MYOF	ADS4647<br>ADS4648	GAATGGCTGGTTGGATGACT<br>ATGAGCCCTCACTTTGCATC	chr10:95195294+95195525	231
MAGEA10	ADS4673<br>ADS4674	TCTCACACACAGGCACACAA<br>CAGGGTCTGCTATGCAGTGA	chrX:150991438+150991638	200
PDLIM1	ADS4677<br>ADS4678	TTCCAGAAAGCAGGCAGATT<br>CAACCAACACCCCAGCTTAT	chr10:97016568+97016723	155
c-FOS	ADS4055<br>ADS4056	GAGCAGTTCCCGTCAATCC<br>GCATTTCGCAGTTCCTGTCT	chr14:74814930+74815094	161
FOS	ADS4679<br>ADS4680	TGTTTTCACCTCTGCCTGTG<br>GTGCGCTAACCTCCATTCTT	chr14:74813381+74813503	122
TRIB1	ADS4685<br>ADS4686	CCACCTCCTACTCCCTCTCC<br>CAGTGCACTCCTCCTTCTCC	chr8:126511548+126511760	212
ZNT8	ADS5256<br>ADS5257	AATCACCAGCACCTCATCCA<br>CCTCTCACTGGTCCCATCTG	chr1:217950708+217950888	181
NEK11	ADS5266<br>ADS5267	GTGTGGTTGCCCCTAGTTTG<br>TCCAGGAAAGCGAGGAGATC	chr3:132228455+132228669	215
EFNA5	ADS5262<br>ADS5263	TTCAACGGCAAGAGGGTTTG<br>GCAAACAGACTCCACAAGCA	chr5:106883724+106883845	122
TFDP2	ADS5264<br>ADS5265	CCCACTTCCTTACCTCTGCA<br>TCTCTTTCTGGGGCTGGTTT	chr3:143318987+143319119	133
COL4A2	ADS5260<br>ADS5261	TGGACATGCTTTGGTCTGGA<br>TGCAGACAACATGCTTCACC	chr13:109936244+109936436	193
APAK13	ADS5258<br>ADS5259	CCTGATTGACATCTGCTGGC<br>AACAAGTAGCTGGCTCGAGT	chr15:83788248+83788439	192
TYW1	ADS5246<br>ADS5247	GGACAGGTTGGCTAGAGTGT<br>ATCCCTTGTGCTTCCGGTAA	chr7:66231490+66231726	237
L3MBTL4	ADS5248<br>ADS5249	TCAAGTGTCTCAGGTCGACC<br>TTTGTATCCCAGTGGCACCT	chr18:6373169+6373360	192
NTM	ADS5250<br>ADS5251	TGGCACATTCCACTGGTTTG<br>TATGTGGTGAGGAGTTGGGG	chr11:131440147+131440392	246
KDM3A	ADS2618<br>ADS2619	ACCTGCTTGGGCCTTATCTT<br>CTAGGCACCAATTCCCAGAA	chr2:86474434+86474628	194

CHD4	ADS2652<br>ADS2653	CATCCACCCCCAGTTTGATA<br>GGCAGCTCTAGGGGGTTAGT	chr12:6587430+6587586	156
NXPH2	ADS2654<br>ADS2655	TATGCTGGGATTGGGGAATA<br>ACATTCCTTACGCTGCCATC	chr2:139252106+139252266	160
PLCL2	ADS2926<br>ADS2927	TCCTTCTGGAATTCCCCTCT<br>AATATCGTCCCCCGATTCAT	chr3:17056207+17056361	154
HPS4	ADS2939<br>ADS2940	GGAGCGGCTCAGAGATACAA<br>AGGCCGCCTCTCTTGATATT	chr22:25197819+25197959	140
ANKRD1	ADS4683<br>ADS4684	GAGGGGAGGACAAGCTAACC<br>AGCTGTCCCCTGACTCTTGA	chr10:92670981+92671109	128
KLHL5	ADS4681<br>ADS4682	AGGAGGTGGAGGGTGTCTCT<br>GAAGCGGATGGGACAATTTA	chr4:38721542+38721678	136
CCNB1	ADS2534<br>ADS2535	GGAGGGAGGAAGGTGAGAAA<br>CTCTCTGGGAAGGGGGTTAC	chr5:68463301+68463446	146
SCNN1A	ADS2937<br>ADS2938	GTTCCTGGGACTGGATGAAA<br>AGGAAGTGCTGAGTCCGAAG	chr12:6350317+6350469	152
IRS2	ADS2924<br>ADS2925	TGCCCTGCTCACACTGTCTA<br>TGTGTGGTTGGCTCTTATGC	chr13:109325328+109325497	169
CDKN1B	ADS2381<br>ADS2382	GGCCTCAGAAGACGTCAAAC<br>AGCCTTCCCCATTGCTACTT	chr12:12871857+12872039	183
CIR1	ADS2930<br>ADS2931	CTAGAGAAGCGGAGGCTGTC<br>TTTCATCCTGCCTCCAAATC	chr2:174968410+174968557	146

A. Oligonucleotide primers used in ChIP.

NAME	PRIMER NUMBER	DIRECTION	SEQUENCE
RT_FOS	ADS1690<br>ADS1691	FORWARD PRIMER<br>REVERSE PRIMER	AGAATCCGAAGGGAAAGGAA<br>CTTCTCCTTCAGCAGGTTGG
RT_ANKRD1	ADS4707<br>ADS4708	FORWARD PRIMER<br>REVERSE PRIMER	AGCCCAGATCGAATTCCGTG<br>ACAGGCGATAAGATGCTCCG
RT_TRIB1	ADS4709<br>ADS4710	FORWARD PRIMER<br>REVERSE PRIMER	GCTACGGCAGCTTCTCTGAT<br>AGTGCAGATGAGGTCTTGGC
RT_GAPDH	ADS2184<br>ADS2185	FORWARD PRIMER<br>REVERSE PRIMER	ACAGTCAGCCGCATCTTCTT<br>TTGATTTTGGAGGGATCTCG
RT_HMBS	ADS2858<br>ADS2859	FORWARD PRIMER<br>REVERSE PRIMER	GAGAAGAATGAAGTGGACCT<br>GAAAGACAACAGCATCATGAG
RT_18S	ADS4005<br>ADS4006	FORWARD PRIMER<br>REVERSE PRIMER	CGGCTACCACATCCAAGGAA<br>GCTGGAATTACCGCGGCT

B. Oligonucleotide primers used in RT-PCR.

NAME	PRIMER NUMBER	SEQUENCE
CYP27C1wt	ADS2782<br>ADS2783	ctag AACATGTTAAT**GTAAACA**AGGAAGCCTG<br>ctag CAGGCTTCCT**TGTTTAC**ATTAACATGTT
CYP27C1mut1	ADS3737<br>ADS3738	ctag AACATGTTA**GG****GTAAACA**AGGAAGCCTG<br>ctag CAGGCTTCCT**TGTTTAC****CC**TAACATGTT
CYP27C1mut2	ADS3739<br>ADS3740	ctag AACATGTTAAT**GTAAACA****GA**GAAGCCTG<br>ctag CAGGCTTC**TC****TGTTTAC**ATTAACATGTT

C. Oligonucleotides used in bandshift experiments.

Supplementary Table S1
